# Srs2 and Mus81–Mms4 Prevent Accumulation of Toxic Inter-Homolog Recombination Intermediates

**DOI:** 10.1371/journal.pgen.1006136

**Published:** 2016-07-07

**Authors:** Kenji Keyamura, Kota Arai, Takashi Hishida

**Affiliations:** Department of Life Science, Graduate School of Science, Gakushuin University, Tokyo, Japan; University of Copenhagen, DENMARK

## Abstract

Homologous recombination is an evolutionally conserved mechanism that promotes genome stability through the faithful repair of double-strand breaks and single-strand gaps in DNA, and the recovery of stalled or collapsed replication forks. *Saccharomyces cerevisiae* ATP-dependent DNA helicase Srs2 (a member of the highly conserved UvrD family of helicases) has multiple roles in regulating homologous recombination. A mutation (*srs2*^*K41A*^) resulting in a helicase-dead mutant of Srs2 was found to be lethal in diploid, but not in haploid, cells. In diploid cells, Srs2^K41A^ caused the accumulation of inter-homolog joint molecule intermediates, increased the levels of spontaneous Rad52 foci, and induced gross chromosomal rearrangements. Srs2^K41A^ lethality and accumulation of joint molecules were suppressed by inactivating Rad51 or deleting the Rad51-interaction domain of Srs2, whereas phosphorylation and sumoylation of Srs2 and its interaction with sumoylated proliferating cell nuclear antigen (PCNA) were not required for lethality. The structure-specific complex of crossover junction endonucleases Mus81 and Mms4 was also required for viability of diploid, but not haploid, *SRS2* deletion mutants (*srs2*Δ), and diploid *srs2*Δ *mus81*Δ mutants accumulated joint molecule intermediates. Our data suggest that Srs2 and Mus81–Mms4 have critical roles in preventing the formation of (or in resolving) toxic inter-homolog joint molecules, which could otherwise interfere with chromosome segregation and lead to genetic instability.

## Author Summary

Homologous recombination (HR) is a DNA-repair mechanism that is generally considered error free because it uses an intact sister chromatid as a template. However, in diploid cells, HR can also occur between homologous chromosomes, which can lead to genomic instability through loss of heterozygosity. This alteration is often detected in genetic disorders and cancer, suggesting that tight control of this process is required to ensure genome stability. Yeast Srs2, conserved from bacteria to humans, plays multiple roles in the regulation of HR. We show here that a helicase-dead mutant of Srs2, *srs2*^*K41A*^, is lethal in diploid cells but not in haploid cells. Expression of Srs2^K41A^ in diploid cells causes inter-homolog joint molecule intermediates to accumulate, and leads to gross chromosomal rearrangements. Moreover, *srs2*Δ *mus81*Δ double mutants have a severe diploid-specific growth defect with accumulation of inter-homolog joint molecules. These data demonstrate that Srs2 and Mus81-Mms4 participate in essential pathways preventing accumulation of inter-homolog recombination intermediates, thereby reducing the risk of genome instability.

## Introduction

Genomes are constantly challenged by endogenous metabolic products or exogenous physical or chemical agents that can generate DNA lesions. When they go unrepaired, these DNA lesions cause stalled replication forks and/or replication-fork collapse, leading to the accumulation of single-stranded DNA (ssDNA) gaps or DNA double-strand breaks (DSBs). Homologous recombination (HR) is a highly conserved DNA-repair mechanism that is essential for the faithful repair of DSBs and has an important role in the repair of post-replicative ssDNA gaps [1–3]. Therefore, dysregulated or incomplete repair by HR can lead to genomic instability, which is a hallmark of cancer.

Rad51 is a central factor in DSB repair by HR. Rad51 forms nucleoprotein filaments on ssDNA tracts generated by 5’ to 3’ ssDNA resection from DSBs. Rad51 filaments mediate strand invasion into homologous DNA duplexes, leading to the formation of D-loops [4,5]. HR intermediates, including D-loops, can enter one of two HR sub-pathways: the synthesis-dependent strand-annealing (SDSA) pathway, which generates non-crossover products, and the canonical DSB repair (DSBR) pathway, which generates crossover or non-crossover products [6,7]. In the SDSA pathway, a newly synthesized ssDNA strand is displaced from the D-loop to anneal to the complementary strand in the original duplex, resulting in a non-crossover outcome with no change to the template DNA [1]. The DSBR pathway involves D-loop extension and annealing of the displaced strand to a second ssDNA tail of the broken duplex, forming a DNA intermediate termed the double Holliday junction. In *Saccharomyces cerevisiae*, several helicases function in crossover control. Srs2 and Mph1 act independently to promote SDSA by processing the HR intermediates downstream of D-loop formation [8–11]. Sgs1, together with Top3 and Rmi1, can dissociate double Holliday junctions to generate non-crossover products, thus preventing crossovers in the DSBR pathway [8,12–14]. Alternatively, double Holliday junctions can be resolved to produce crossover or non-crossover products by structure-specific endonucleases, such as the Mus81–Mms4 complex, the Slx1–Slx4 complex, and Yen1 [15–17].

Srs2 is a member of the highly conserved UvrD family of helicases that have 3’ to 5’ helicase activity [18,19]. A mutant allele of *SRS2* was first isolated as a suppressor of the radiation sensitivity associated with *rad6* and *rad18* mutants, which are defective in post-replication repair [20–22]. In addition, mutants of *SRS2* have a hyper-recombination phenotype characterized by crossover events [8,23,24]. Srs2 interacts with a sumoylated form of the DNA replication clamp, proliferating cell nuclear antigen (PCNA), which recruits Srs2 to DNA replication forks, preventing HR [25,26]. Thus, Srs2 is an anti-recombinase that prevents inappropriate HR at the replication fork and preferentially facilitates post-replication repair. These data are consistent with the fact that Srs2 disassembles the Rad51 filament and unwinds synthetic D-loop structures *in vitro* [27–29]. In addition to its role as an anti-recombinase, Srs2 binding to sumoylated PCNA blocks synthesis-dependent elongation of the invading strand within a D-loop structure at a stalled replication fork, limiting the frequency of crossover events [29]. Moreover, Srs2 promotes the SDSA pathway during mitotic DSB repair by removing the Rad51 filament from the second end of the DSB, and/or by facilitating the dissociation of the invading strand from the D-loop [30–32]. Phosphorylation of Srs2 by cyclin-dependent kinase 1 (Cdk1) stimulates the SDSA pathway [33]. Taken together, these observations suggest that Srs2 has two distinct functions in HR; it prevents unscheduled recombination by inhibiting Rad51-dependent formation of joint molecules and it promotes efficient DSB repair by the SDSA pathway.

During HR in diploid cells, sister chromatids are the preferred templates for HR-mediated repair (inter-sister HR), but homologous chromosomes can also be used to restore the broken DNA (inter-homolog HR), although much less efficiently. Because sister chromatids are identical, inter-sister HR is genetically silent. By contrast, the use of homologous chromosomes as repair templates has important consequences for genetic stability, and loss of heterozygosity is a frequent outcome [34]. The frequency of loss of heterozygosity is high in cancerous and aged cells, which has raised interest in dissecting the mechanisms of HR [35]. The HR process has to be tightly controlled to protect against genetic instability, but little is known about the relative contributions of each HR pathway to the processing of the two classes of recombination intermediate, involving either sister chromatids or homologs.

Our experiments were designed to explore the role of Srs2 in haploid and diploid cells by phenotypic characterization of a number of *srs2* mutants as a function of cell ploidy. The Srs2 helicase-deficient mutant (*srs2*^*K41A*^) caused diploid-specific lethality. This lethality was suppressed by deletion of *RAD51*, but was independent of the phosphorylation and sumoylation of Srs2 and of its interaction with sumoylated PCNA. Expression of Srs2^K41A^ in diploid cells led to a specific increase in G_2_/M-arrested cells, more abundant inter-homolog joint molecules and increased gross chromosomal rearrangements, such as chromosome loss and translocations. *srs2*Δ *mus81*Δ double mutants also demonstrated a severe, diploid-specific growth defect, with the concomitant accumulation of joint molecules. These results suggest that the mechanisms of processing inter-sister and inter-homolog joint molecules differ significantly. We propose that Srs2 and Mus81–Mms4 have critical roles in processing inter-homolog joint molecules, which could otherwise interfere with chromosome segregation and lead to genetic instability.

## Results

### Helicase-dead *srs2*^*K41A*^ is lethal in diploid yeast

**Fig 1:**
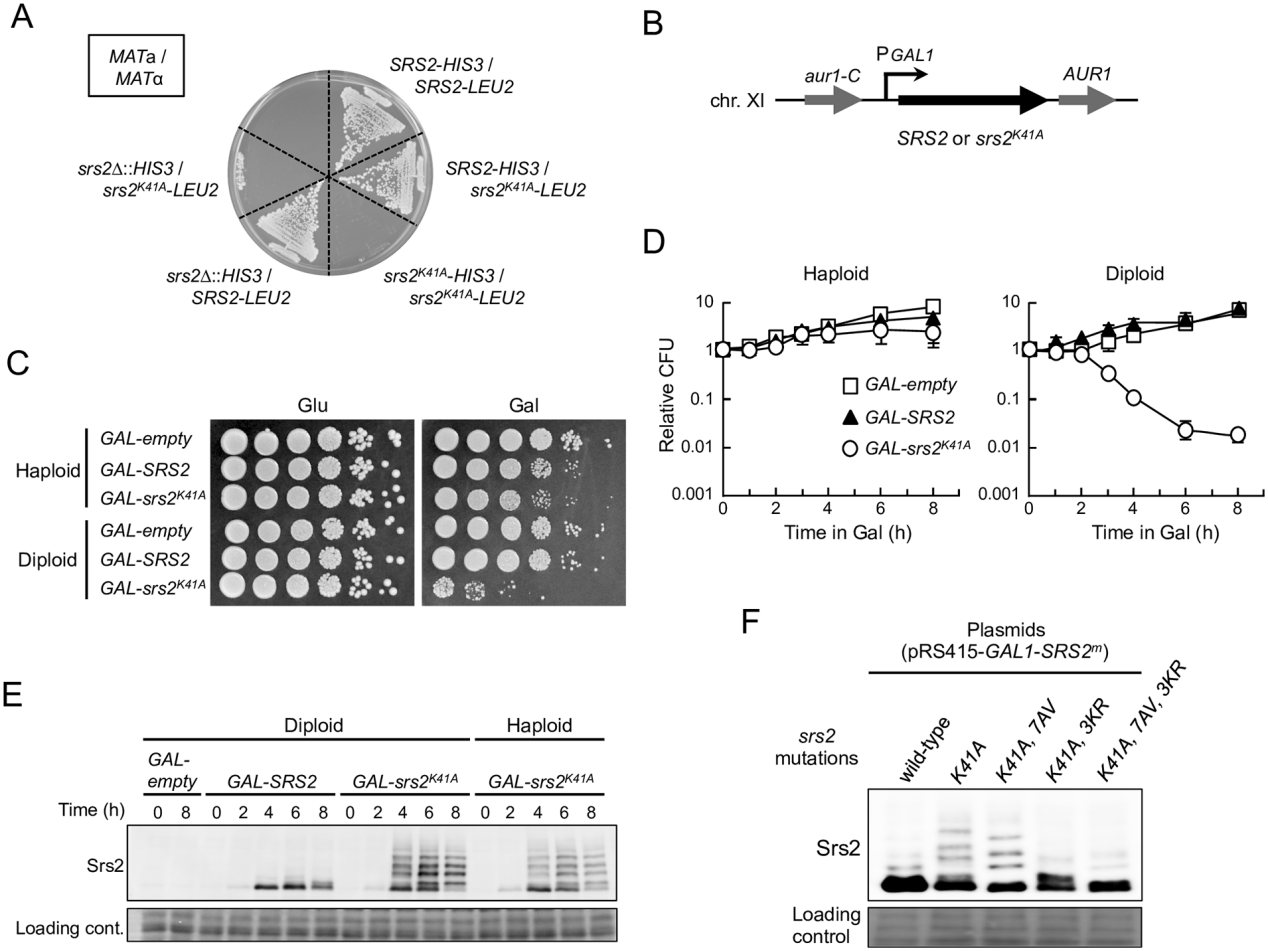
Physiological expression of Srs2^K41A^ causes diploid-specific lethality. (A) *MAT*a haploid cells were mated with *MAT*α cells on YPD plates, generating *MAT*a/*MAT*α diploid cells. The indicated diploid cells were then selected at 30°C for 3 days on SC+Glu plates lacking histidine and leucine (SC+Glu-His-Leu). (B) A DNA fragment with a galactose-inducible promoter and wild-type *SRS2* (*GAL-SRS2*), *srs2*^*K41A*^ (*GAL-srs2*^*K41A*^), or no insertion (*GAL-empty*) was integrated into the *AUR1* locus of *srs2Δ* cells. In all *GAL*-promoter-integrated haploid and diploid strains, the endogenous copy of *SRS2* was deleted to eliminate the expression of wild-type Srs2 from its own locus. (C) Cells grown in YPD medium were diluted and spotted onto YPD plates and YPR + 0.02% galactose plates. These plates were incubated at 30°C for 3 days. (D) For quantitative assays, cells grown to early logarithmic phase in YPD were transferred to YPR containing 0.02% galactose for further incubation, and then plated on YPD to determine the plating efficiency. Cell viability is represented as relative colony-forming units (CFU), such that CFU = 1 at 0 h. Data were obtained from at least three independent experiments. Error bars indicate the standard error for each data point. *GAL-empty* (open squares); *GAL-SRS2* (filled triangles); *GAL-srs2*^*K41A*^ (open circles). (E) The indicated haploid and diploid strains were grown at 30°C in YPR + galactose (0.02%) medium, and cells were harvested at the indicated time points. Protein extracts were prepared and separated by 6% SDS-PAGE, followed by western blotting with anti-Srs2 antibodies. (F) The *srs2*Δ diploid strains carrying the indicated plasmids were grown in SC+Glu-Leu and then transferred into SC-Leu (2% raffinose + 0.2% galactose) medium for 6 h to induce Srs2. Protein extracts were prepared and separated by 6% SDS-PAGE, followed by western blotting with anti-Srs2 antibodies.

A previous study showed that *srs2*Δ diploid cells are more sensitive to methyl methanesulfonate (MMS) than *srs2*Δ haploid cells [21,36]. This ploidy-specific sensitivity to MMS is thought to reflect lethal outcomes of inter-homolog HR events in the absence of wild-type Srs2. To understand the role of Srs2 in inter-homolog HR, we constructed four mutants of *srs2*: *srs2*^*K41A*^ lacks helicase activity [37], *srs2*^*7AV*^ cannot undergo Cdk1-dependent phosphorylation [38,39], *srs2*^*3KR*^ cannot undergo sumoylation [40], and *srs2*^Δ*SIM*^ lacks the protein motif that mediates interaction with sumoylated PCNA [26]. These *srs2* mutants and wild-type *SRS2* were expressed in yeast from low-copy centromeric (pRS415_*LEU2*) plasmids under control of the *SRS2* promoter. The plasmids were introduced into *srs2*Δ haploid or diploid cells and selected on SC+Glucose medium lacking leucine (SC+Glu-Leu). In this initial screen, no diploid colonies expressing Srs2^K41A^ were detected (Table 1), suggesting that *srs2*^*K41A*^ could be lethal or could block growth of *srs2*Δ diploid cells. To test this possibility, an *srs2*^*K41A*^ allele was integrated at the *SRS2* genomic locus of haploid yeast. The integrating cassette included downstream *HIS3* or *LEU2* selectable markers (*srs2*^*K41A*^_*HIS3* or *srs2*^*K41A*^_*LEU2*). The endogenous *SRS2* allele in a haploid strain was also linked to *HIS3* or *LEU2* selectable markers as a control (*SRS2*_*HIS3* or *SRS2*_*LEU2*). A *MAT*α strain carrying *srs2*^*K41A*^_*LEU2* was crossed to *MAT*a strains bearing *srs2*^*K41A*^_*HIS3*, *SRS2*_*HIS3*, or *srs2*Δ::*HIS3*. Diploids from these crosses were selected for growth on SC+Glu medium lacking histidine and leucine. As shown in Fig 1A, the *srs2*^*K41A*^/*srs2*Δ heterozygotes and *srs2*^*K41A*^/*srs2*^*K41A*^ homozygotes did not grow on the selection medium, whereas heterozygous *srs2*^*K41A*^/*SRS2* diploids exhibited normal growth. This demonstrates that *srs2*^*K41A*^ mutants are lethal in diploids.

**Table 1:** *srs2*^*K41A*^ is lethal in diploids, but not in haploids.

Plasmids	Growth of transformants on SC+Glu-Leu plates	
	Haploid *srs2*Δ	Diploid *srs2*Δ
pRS415; vector	+	+
pRS415-*SRS2*	+	+
pRS415-*srs2*^*K41A*^	+	-
pRS415-*srs2*^*7AV*^	+	+
pRS415-*srs2*^*3KR*^	+	+
pRS415-*srs2*^Δ*SIM*^	+	+
pRS415-*srs2*^*K41A*,*7AV*^	+	-
pRS415-*srs2*^*K41A*,*3KR*^	+	-
pRS415-*srs2*^*K41A*,*7AV*,*3KR*^	+	-

The *srs2*Δ haploid and diploid strains transformed with each of the pRS415 derivatives were incubated at 30°C for 3 days on SC+Glu-Leu plates. The pRS415-based vectors contain the *SRS2* alleles under the control of endogenous *SRS2* promoter. +, viable colonies were detected at similar levels to those with an empty-vector control; -, no colonies were detected.

### Srs2^K41A^ differentially inhibits growth of haploid and diploid yeast cells

To investigate why *srs2*^*K41A*^ is lethal in diploid cells, Srs2^K41A^ and wild-type Srs2 were expressed under the control of the inducible *GAL1* promoter from a single-copy integrated allele at the chromosomal *AUR1* locus of *srs2*Δ diploid and haploid cells (Fig 1B). Hereafter, these strains are referred to as *GAL-srs2*^*K41A*^ and *GAL-SRS2*, respectively. A *GAL-empty* strain (essentially the same as an *srs2Δ* strain) was constructed in a similar manner, as an additional control. The resultant haploid and diploid strains grew normally in 2% glucose-containing medium (YPD) (Fig 1C and S1A Fig), enabling the effect of conditional expression of Srs2^K41A^ and Srs2 to be investigated.

To determine the level of expression of Srs2 in this experimental system, *GAL-SRS2* diploid cells were grown for 6 h in the presence of 2% raffinose medium (YPR) and various concentrations of galactose, and whole-cell extracts were prepared and analyzed by immunoblotting with an antibody to Srs2. The results revealed that Srs2 protein was absent in cells grown in YPD or YPR, and that the abundance of Srs2 increased with increasing galactose concentration (S1B Fig). Control experiments established that *GAL-SRS2* diploid cells grew normally in the presence of 0.02% galactose, but poorly in the presence of 0.2% galactose, because of high overexpression of Srs2 (Fig 1C and S1C Fig), as previously reported [36]. In addition, expression of Srs2^K41A^, but not wild-type Srs2, inhibited growth (despite the presence of the chromosomal *SRS2*^+^ allele) when moderately expressed in the presence of 0.05% galactose, whereas similar growth defects were not observed in the presence of 0.02% galactose (S1D Fig). Thus, *srs2*^*K41A*^ is essentially a dominant-negative allele, and its dominancy is dependent on the ratio of wild-type Srs2 to Srs2^K41A^. We conclude that expression of Srs2 from the *GAL1* promoter in the presence of 0.02% galactose generates a physiologically-relevant protein level, and, for the remainder of this study, cells carrying *GAL1* promoter-driven expression strains were grown in YPD or YPR to repress Srs2 expression, and in YPR medium containing 0.02% galactose to induce Srs2.

### Expression of Srs2^K41A^ reduces viability of the *srs2Δ* diploid strain

To examine whether *GAL-srs2*^*K41A*^ diploid cells could recover from growth arrest in galactose-containing medium, cells transiently grown in the presence of 0.02% galactose were transferred back to glucose-containing medium to determine the plating efficiency. The plating efficiency of *GAL-srs2*^*K41A*^ diploids decreased rapidly with >3 h incubation in the presence of galactose, whereas no significant effect on growth was observed for *GAL-srs2*^*K41A*^ haploid cells, or *GAL-empty* and *GAL-SRS2* haploid or diploid cells, even after incubation for 8 h in 0.02% galactose (Fig 1D). These data show that a physiological level of Srs2^K41A^ reduces viability of diploid cells, but not haploid cells.

### The lethality of *GAL-srs2*^*K41A*^ diploids does not depend on post-translational modification

In the course of these studies, Srs2^K41A^ isolated from haploid and diploid cells was observed as multiple slow-migrating protein species on SDS-PAGE when cells were grown in the presence of 0.02% galactose (Fig 1E). Because Srs2 is phosphorylated and sumoylated in response to DNA damage [33,38,39], we postulated that the slower-migrating forms of Srs2^K41A^ protein are phosphorylated and/or sumoylated isoforms of the protein. To test this hypothesis, plasmids that expressed Srs2^K41A^, Srs2^K41A,7AV^, Srs2^K41A,3KR^, and Srs2^K41A,7AV,3KR^ from the *GAL1* promoter were introduced into *srs2*Δ diploid cells. Each strain was grown to early logarithmic phase in glucose medium and transferred to galactose medium, and protein extracts were prepared and analyzed by western blot with an antibody to Srs2. This analysis revealed that Srs2^K41A,7AV^, which lacked phosphorylation sites, existed as three sumoylated isoforms that moved slightly faster than modified isoforms of Srs2^K41A^ on electrophoresis (Fig 1F). Srs2^K41A,3KR^, which lacked sumoylation sites, existed as phosphorylated isoforms (Fig 1F). As expected, *srs2*^K41A,7AV,3KR^, in which all phosphorylation and sumoylation sites had been mutated, resulted in a considerable reduction in expression of modified isoforms of Srs2 (Fig 1F). These results indicate that Srs2^K41A^ can be sumoylated and phosphorylated in the absence of DNA damage. To determine whether these modifications of Srs2^K41A^ affected diploid-specific lethality, yeast *CEN/ARS* plasmids (in which *srs2*^*K41A*^, *srs2*^*K41A*,*7AV*^, *srs2*^*K41A*,*3KR*^, and *srs2*^*K41A*,*7AV*,*3KR*^ were under the control of the endogenous *SRS2* promoter) were constructed and transformed into the *srs2*Δ diploid strain. The result showed that no *srs2*Δ transformants expressing Srs2^K41A^ or its derivatives were viable (no colonies were detected) (Table 1), indicating that neither phosphorylation nor sumoylation is required for the lethal effects of Srs2^K41A^ in diploid yeast.

### *GAL-srs2*^*K41A*^ diploids arrest at G_2_/M

**Fig 2:**
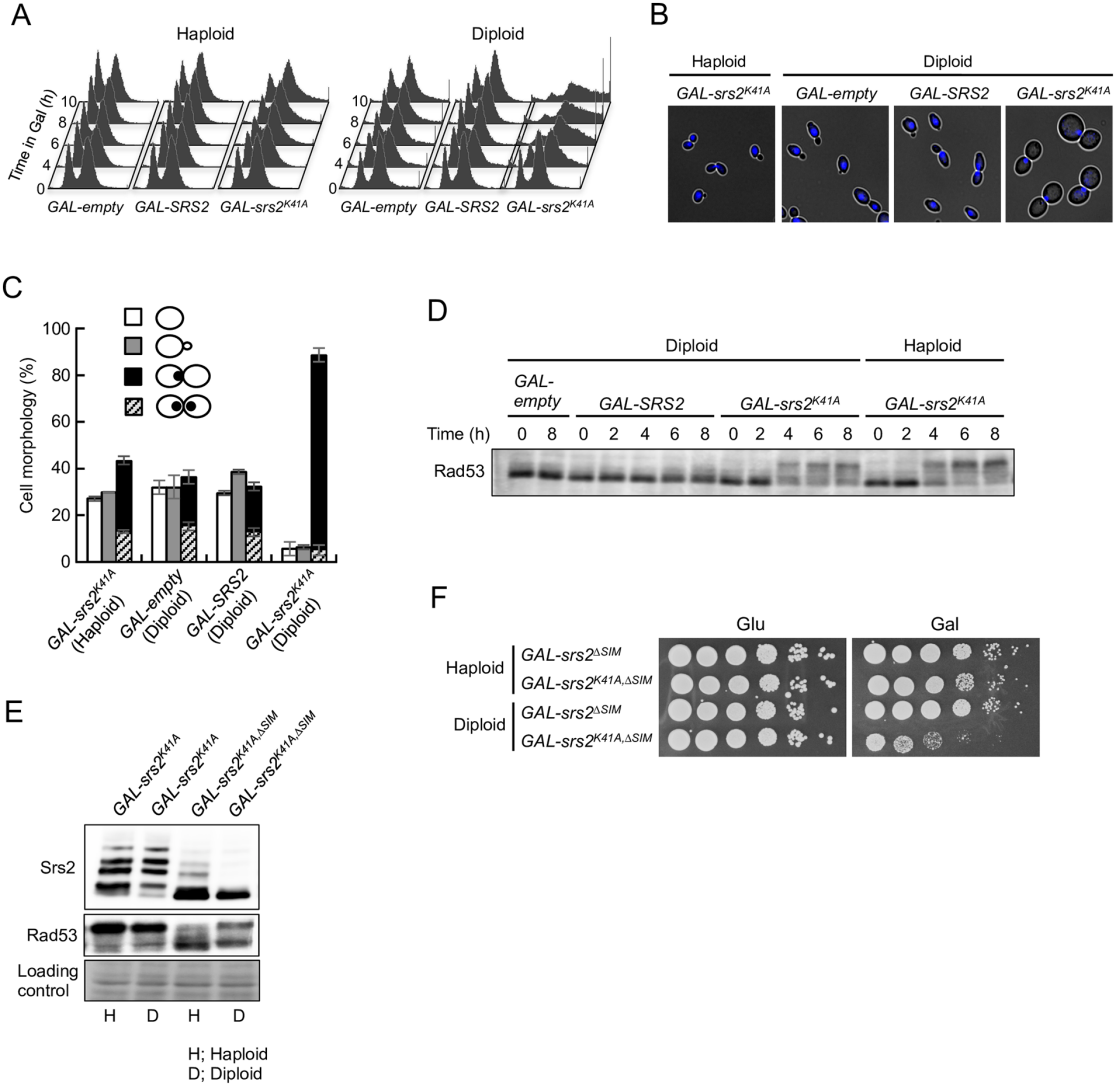
Expression of Srs2^K41A^ causes G_2_/M arrest in diploids but not in haploids. (A) Asynchronous cells were grown at 30°C in YPR + galactose (0.02%), and samples were collected at the indicated time points. DNA content was measured by FACS. (B and C) Cells grown in YPR + galactose (0.02%) medium for 8 h were fixed with ethanol and stained with 4,6-diamidino-2-phenylindole (DAPI) to visualize the DNA. Representative morphology observed after transfer to the YPR + galactose (0.02%) medium for 8 h is shown in (B). Cells with no bud (G_1_ phase), cells with small bud (S phase), and large-budded cells with one or two nuclei at the bud neck (G_2_/M phase) were scored (C). The results represent the averages of at least three independent measurements. Error bars indicate the standard error for each data point. (D) The DNA-damage checkpoint is activated in *srs2*^*K41A*^ haploid and diploid cells. The indicated haploid and diploid strains were grown in YPD medium. Cells were transferred to YPR + 0.02% galactose to induce Srs2 expression and then cultured at 30°C for the indicated times. Protein extracts were prepared and separated by 6% SDS-PAGE, followed by western blotting with anti-Rad53 antibody. (E) *GAL-srs2*^*K41A*^ and *GAL-srs2*^*K41A*,Δ*SIM*^ haploid and diploid cells were grown in YPD medium. Cells were transferred to YPR + 0.02% galactose to induce Srs2 expression and then cultured at 30°C for 6 h. Protein extracts were prepared and separated by 6% SDS-PAGE, followed by western blotting with anti-Srs2 or Rad53 antibodies. (F) Cells grown in YPD were diluted and spotted onto YPD plates (Glu) and YPR + 0.02% galactose plates (Gal). These plates were incubated at 30°C for 3 days.

To learn more about how *srs2*^*K41A*^ kills diploid yeast cells, the cell-cycle distribution and cell morphology of *GAL-srs2*^*K41A*^ cells were investigated in haploid and diploid cells. Cells were grown to early logarithmic phase in the presence of glucose, transferred to YPR containing 0.02% galactose, and then analyzed by flow cytometry. In *GAL-empty* and *GAL-SRS2* haploids and diploids, cell-cycle progression was not significantly altered by galactose induction (Fig 2A). However, *GAL-srs2*^*K41A*^ diploids, but not haploids, showed apparent cell-cycle arrest at G_2_/M after induction of Srs2^K41A^. The 4C peak appeared to broaden with prolonged incubation of cells in the presence of 0.02% galactose (Fig 2A). Similar effects have been observed after extended treatment with nocodazole, a microtubule-depolymerizing drug that causes G_2_/M arrest [41]. Consistent with this interpretation, approximately 80% of *GAL-srs2*^*K41A*^ diploids assumed the characteristic morphology of G_2_/M arrest, which involves large-budded cells with one nucleus at the bud neck and a short spindle (Fig 2B and 2C and S2 Fig). These results suggest that, in diploids, Srs2^K41A^ causes cell-cycle arrest after bulk DNA synthesis is complete.

### The lethality of *GAL-srs2*^*K41A*^ diploids is not dependent on its interaction with sumoylated PCNA

The checkpoint protein kinase Rad53 is phosphorylated and activated in response to DNA damage and replication stress. As shown in Fig 2D, phosphorylated Rad53 was detected in galactose-induced *GAL-srs2*^*K41A*^ diploid and haploid cells, but not in *GAL-SRS2* cells. Previous studies showed that the protein product of *srs2*^Δ*SIM*^, which cannot interact with sumoylated PCNA, undergoes dramatically less sumoylation *in vivo* [40], and *srs2*^Δ*SIM*^ mutation suppresses the replication defects associated with overexpression of Srs2 in haploid cells [42]. In our study, the phenotypes of *GAL-srs2*^*K41A*,Δ*SIM*^ diploid and haploid cells were examined. Rad53 phosphorylation and Srs2 sumoylation (and phosphorylation) were significantly reduced at 6 h after *GAL-srs2*^*K41A*,Δ*SIM*^ haploid cells were transferred to 0.02% galactose, compared with levels in *GAL-srs2*^*K41A*^ haploid cells (Fig 2E). By contrast, substantial Rad53 phosphorylation was still observed in *GAL-srs2*^*K41A*,Δ*SIM*^ diploid cells, although Srs2 phosphorylation and sumoylation were strongly reduced compared with levels in *GAL-srs2*^*K41A*^ diploid cells (Fig 2E). In addition, *GAL-srs2*^*K41A*,Δ*SIM*^ diploids, but not haploids, had severe growth defects (Fig 2F). These results indicate that the *srs2*^*K41A*^ lethality in diploid cells is not associated with activation of the DNA damage checkpoint through its interaction with sumoylated PCNA.

### The *srs2*^*K41A*^ lethality in diploids is dependent on homologous recombination

**Fig 3:**
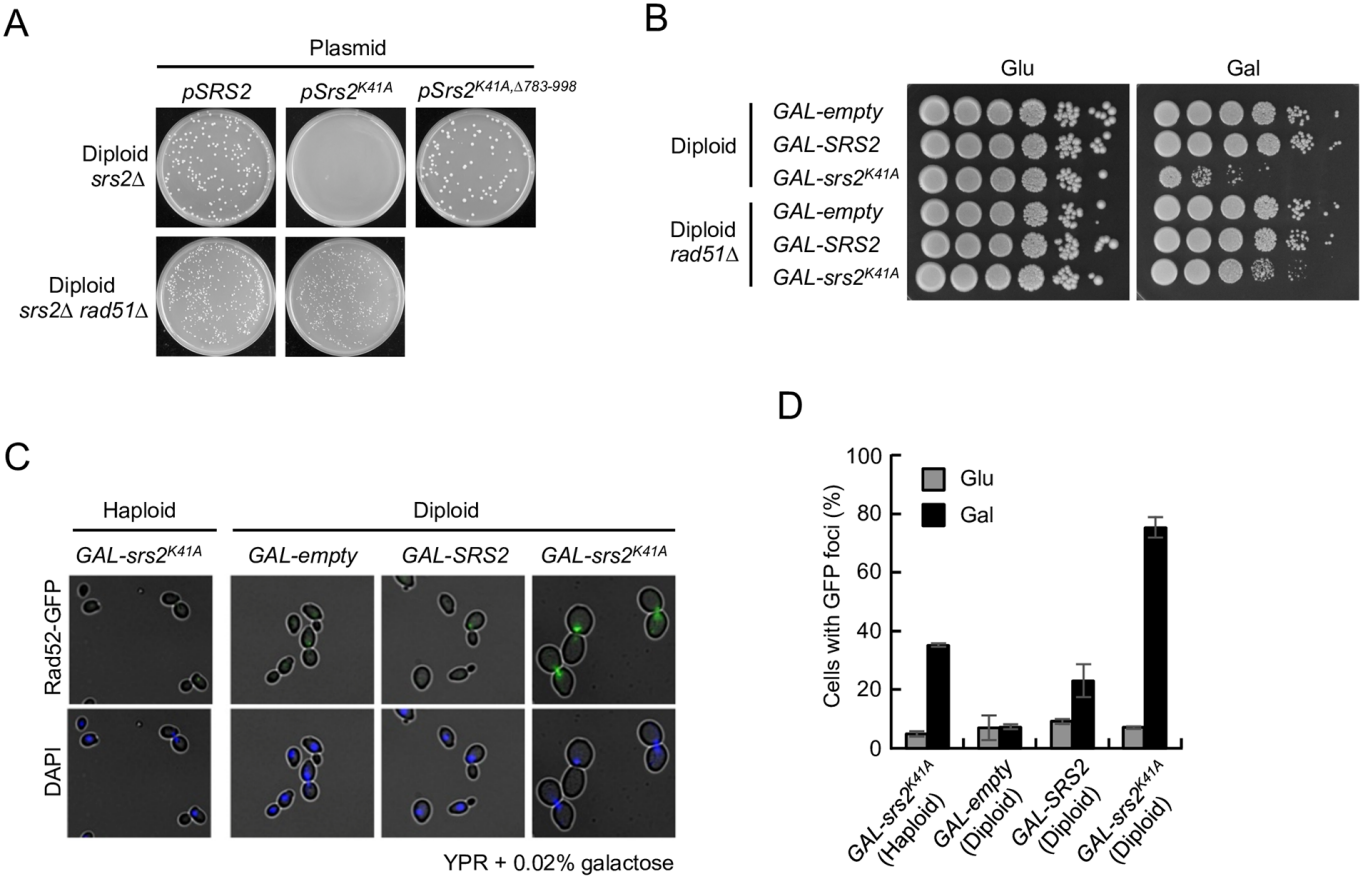
Expression of Srs2^K41A^ causes toxic recombination intermediates to accumulate in diploids. (A) *srs2*Δ and *srs2*Δ *rad51*Δ diploid cells were transformed with each of the pRS415 derivatives bearing *SRS2*, *srs2*^*K41A*^, or *srs2*^*K41A*,Δ*783–998*^, and the cells were incubated at 30°C for 3 days on SC+Glu-Leu plates. (B) Cells grown in YPD were diluted and spotted onto YPD plates (Glu) and YPR + 0.02% galactose plates (Gal). These plates were incubated at 30°C for 3 days. (C) Images of cells with Rad52-GFP foci in Srs2^K41A^-expressing diploid cells. The indicated strains were grown at 30°C for 8 h in YPD (Glu) or YPR + 0.02% galactose (Gal). Cells were collected, stained with 4,6-diamidino-2-phenylindole (DAPI), and examined by fluorescence microscopy. (D) Quantitation of cells with Rad52-GFP foci in Srs2^K41A^-expressing diploid cells. Error bars indicate the standard error for each data point. Representative images of Rad52-GFP foci and DAPI staining are shown in (C).

A well-characterized role of Srs2 is that of anti-recombinase, and in this context Srs2 dismantles Rad51 nucleofilaments on ssDNA [27,28]. Toxic HR intermediates might, therefore, accumulate in *srs2*^*K41A*^ diploid cells, which could explain the ploidy-specific lethality of this allele. Consistent with this hypothesis, *rad51Δ srs2*Δ diploid strains expressing Srs2^K41A^ from a plasmid vector were viable (Fig 3A). Similarly, the growth inhibition of *GAL-srs2*^*K41A*^ diploids in the presence of 0.02% galactose was suppressed by the *rad51*Δ mutation (Fig 3B). Moreover, *srs2*Δ diploid cells expressing Srs2^K41A,Δ783–998^, which lacks the Rad51 interaction domain in Srs2 [28], were also viable (Fig 3A). Taken together, these results indicate that the lethality of *srs2*^*K41A*^ in diploids is associated with Rad51-dependent HR in diploids.

### Srs2^K41A^ causes Rad52-GFP foci to accumulate in diploid cells

Rad52 nuclear focus formation is an indication of HR *in vivo*, and many mutants with genome-maintenance defects have increased numbers of Rad52 foci compared with their wild-type counterparts [43]. The frequency of spontaneous Rad52 foci was, therefore, quantified in *GAL-srs2*^*K41A*^ cells and appropriate control cells expressing GFP-tagged Rad52 from the endogenous *RAD52* genomic locus. Few Rad52-GFP foci were observed when cells were grown in glucose-containing medium (Fig 3C and 3D). However, after 8 h incubation in 0.02% galactose, Rad52-GFP foci were markedly increased in *GAL-srs2*^*K41A*^ diploids compared with *GAL-SRS2* diploid and *GAL-srs2*^*K41A*^ haploid cells, and most of the foci occurred in large-budded cells with a single nucleus (Fig 3C and 3D). These findings suggest that *GAL-srs2*^*K41A*^ diploid cells accumulate HR intermediates at a much higher frequency than *GAL-srs2*^*K41A*^ haploid cells.

### Inter-homolog joint molecules accumulate in *GAL-srs2*^*K41A*^ diploid cells

**Fig 4:**
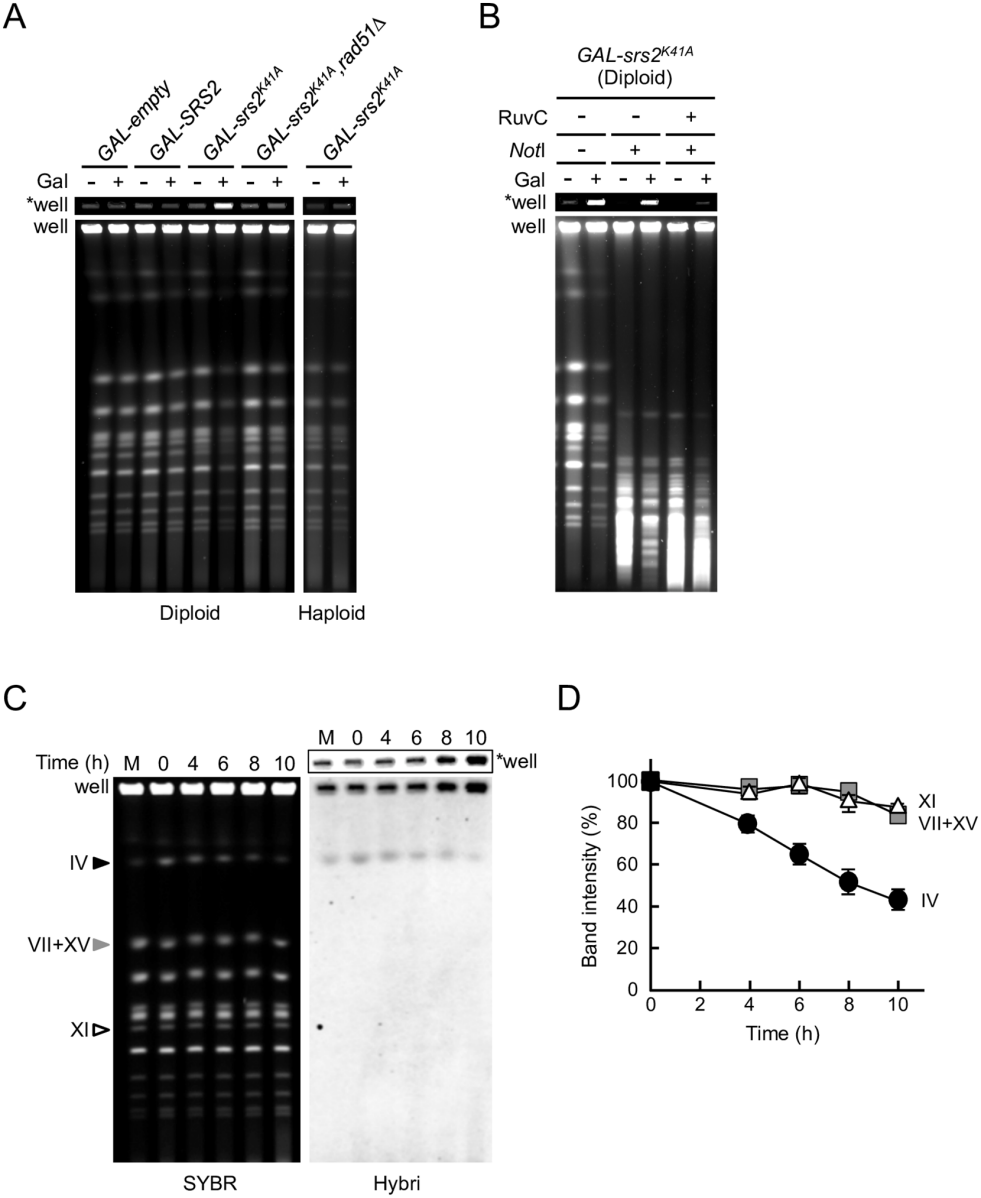
Inter-homolog joint molecules containing Holliday junction-like structures accumulate in *GAL-srs2*^*K41A*^ diploid cells. (A–D) PFGE analysis of chromosomal DNA from Srs2^K41A^-expressing diploid cells. (A) The indicated haploid and diploid strains were grown in YPR or YPR + 0.02% galactose for 8 h. Chromosomal DNA was separated by PFGE and detected by staining with SYBR green. (B) *GAL-srs2*^*K41A*^ diploid cells were grown at 30°C for 4 h in YPR or YPR + 0.02% galactose medium. DNA was isolated using agarose-gel blocks, digested with *Not*I or *Not*I + RuvC, and subjected to PFGE. (C) The *GAL-srs2*^*K41A*^ disome IV strain was transferred to SC+Raffinose-His+G418 medium containing 0.5% galactose to induce the expression of Srs2^K41A^. Chromosomal DNA was analyzed by PFGE, followed by hybridization with a chr. IV probe. “SYBR” indicates a SYBR-green-stained gel, “Hybri” indicates a Southern analysis with the chr. IV probe, and “M” indicates haploid DNA as a size marker. (D) The band intensities of chromosomes IV (circle), VII+XV (square), and XI (triangle) detected by staining the gel were quantified and are shown relative to 100% at time 0. Error bars indicate the standard error for each data point. “*well” indicates an image at the well of the gel shown at low exposure.

To test directly whether Srs2^K41A^ caused joint molecules to accumulate in *srs2*Δ diploids, diploid cells were incubated for 8 h in YPR medium with or without 0.02% galactose, harvested and used to obtain chromosomal DNA, which was analyzed by pulsed-field gel electrophoresis (PFGE). In *GAL-srs2*^*K41A*^ diploid cells, the DNA signal corresponding to chromosomes that entered the gel decreased after induction in galactose-containing medium, and most of the DNA failed to migrate out of the well of the gel (Fig 4A). The non-migratory DNA appeared by 4 h after induction in galactose-containing medium (S3A Fig). By contrast, non-migratory DNA was not observed when DNA from galactose-induced *GAL-SRS2* and *GAL-empty* diploid cells or *GAL-srs2*^*K41A*^ haploid cells was analyzed by PFGE (Fig 4A). Moreover, accumulation of non-migratory DNA in *GAL-srs2*^*K41A*^ diploid cells was suppressed by *rad51*Δ (Fig 4A). These results suggest that Rad51 and Srs2^K41A^ collaborate in diploid cells to generate DNA structures that are not able to migrate out of the well during PFGE. In this context, it should be noted that the *rad51Δ* mutation did not suppress Rad53 activation in *GAL-srs2*^*K41A*^ and *GAL-srs2*^*K41A*,*ΔSIM*^ diploid cells under the same conditions (S3B Fig), suggesting that joint molecules *per se* are not direct signals for Rad53 activation.

To characterize the chromosomal structures that accumulated in *GAL-srs2*^*K41A*^ diploid cells, chromosomal DNA samples were digested with the rare-cutter restriction endonuclease *Not*I prior to PFGE. Although *Not*I digests yeast chromosomes into multiple large and small fragments, the intensity of the DNA signal in the wells did not change significantly after digestion with *Not*I (Fig 4B). This observation suggested that *GAL-srs2*^*K41A*^ diploid cells accumulated branched DNA structures, which were enriched even after digestion with *Not*I. To test this possibility, *Not*I-digested or non-digested chromosomal DNA samples were digested with purified RuvC from *Escherichia coli*. RuvC is a highly specific endonuclease that resolves Holliday junctions, although it also cleaves three-stranded junctions and nicked Holliday junctions [44,45]. The results showed that the action of RuvC released *Not*I-digested chromosomal fragments into the PFGE gel (Fig 4B), whereas non-migratory chromosomal DNA without *Not*I treatment was hardly resolved by RuvC (S3C Fig). *Not*I digestion could conceivably facilitate the formation of catalytically competent joint molecule configurations for RuvC cleavage, since junction incision by RuvC is dependent on configuration [46,47]. These results suggest that RuvC-cleavable joint molecules accumulate in *GAL-srs2*^*K14A*^ diploid cells.

To further examine whether the DNA structures in *GAL-srs2*^*K14A*^ diploid cells were products of inter-homolog HR, an *srs2*Δ haploid strain that carried an additional copy of chromosome IV (henceforth known as the *srs2*Δ disome) was constructed. Notably, no colonies were obtained when Srs2^K41A^ was expressed from a plasmid vector in *srs2*Δ disomes, but the growth defect was rescued by deletion of *RAD51* (S4A Fig). These suggest that the additional copy of a donor sequence (homologous chromosome) is a cause of the lethality of *srs2*Δ disomes expressing Srs2^K41A^, and that the growth defect of *GAL-srs2*^*K14A*^ disomes is the result of Rad51-dependent HR. Similar experiments were performed in an *srs2*Δ disome in which *GAL-srs2*^*K41A*^ was integrated at the chromosomal *AUR1* locus (henceforth known as the *GAL-srs2*^*K41A*^ disome). The *GAL-srs2*^*K41A*^ disome strain failed to grow in the presence of galactose, whereas the haploid control strain grew normally under same conditions (S4B Fig). In PFGE analysis, the *GAL-srs2*^*K41A*^ disome strain, but not *GAL-empty* and *GAL-SRS2* disome strains, showed a specific loss of signal corresponding to chromosome IV in galactose-induced cells, whereas no other chromosomes were similarly affected (Fig 4C and 4D and S4C Fig). This conclusion was confirmed by Southern blotting with a probe for chromosome IV, which showed a reduction in hybridization signal in the gel and augmentation of the hybridization signal in the well during Srs2^K41A^ expression (Fig 4C). These results suggest that inter-homolog joint molecules accumulate in *GAL-srs2*^*K41A*^ diploid and disome cells.

### Srs2^K41A^ induces chromosomal instability in diploid cells

**Fig 5:**
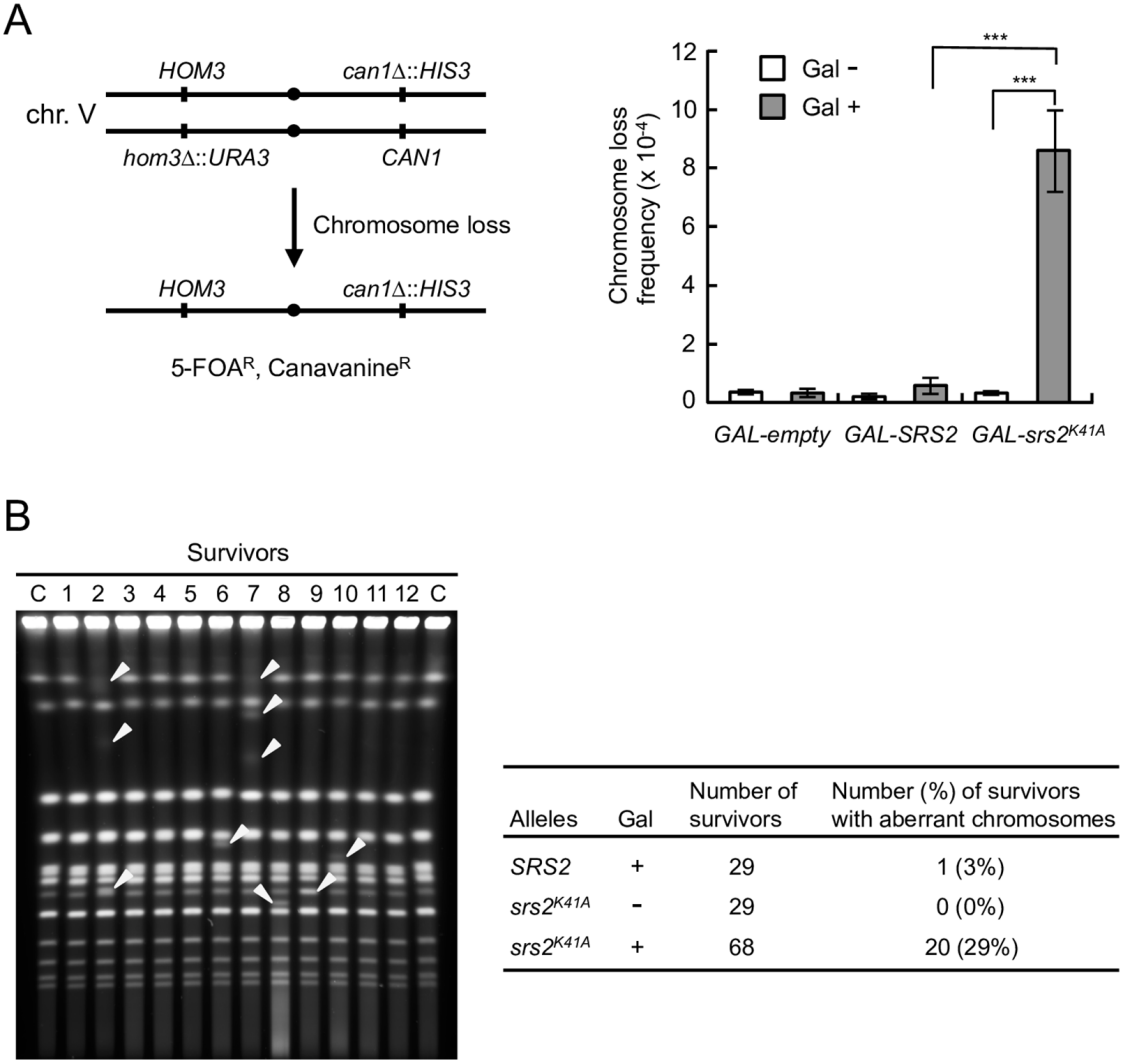
Expression of Srs2^K41A^ increases genome instability in diploids. (A) Schematic representation of the chromosome V homolog used to monitor chromosome loss (left panel). Clones resistant to both 5-fluoroorotic acid (5-FOA) and canavanine (5-FOA^R^ Can^R^) arise because of loss of the entire chromosome with the markers. *GAL-empty*, *GAL-SRS2*, and *GAL-srs2*^*K41A*^ strains were cultured in glucose medium, after which they were placed on YPR or YPR + 0.02% galactose medium for 8 h to induce Srs2 or Srs2^K41A^. The frequency of loss of chr. V was calculated as described in Materials and Methods (right panel). Error bars indicate the standard error for each data point. *** indicates a p value <0.005, calculated using a Student’s two-tailed t test. (B) The indicated cells were grown for 8 h in YPR or YPR + 0.02% galactose medium. The cultures were diluted appropriately and spread onto YPD plates. Chromosomal DNA from the obtained colonies (the survivors) was analyzed using PFGE. The percentage of survivors with aberrant chromosomes is indicated. A representative gel image is shown in the left panel, and abnormal chromosome bands are indicated with arrowheads.

Our results led to the hypothesis that unresolved joint molecules form in *srs2*^*K41A*^ diploid cells, leading to chromosomal instability and cell death. To test this hypothesis, the frequency of loss of a pair of chromosome V homologs marked with *CAN1* on the right arm and *URA3* on the left arm was calculated and compared in *GAL-SRS2* and *GAL-srs2*^*K41A*^ diploid cells (Fig 5A, left panel) [48]. The observed frequency of chromosome loss in galactose-induced *GAL-srs2*^*K41A*^ diploid cells was 15-fold higher than in galactose-induced *GAL-SRS2* diploid cells (Fig 5A, right panel), suggesting that *GAL-srs2*^*K41A*^ diploid cells have a defect in chromosome segregation, which leads to a high rate of aneuploidy. Indeed, this result probably underestimated the chromosome-loss frequencies in galactose-induced *GAL-srs2*^*K41A*^ diploid cells because it only detected aneuploid cells that remained viable after re-plating on glucose-containing medium. To directly investigate genomic integrity, chromosomal DNA was isolated from surviving cells and analyzed by PFGE. Chromosomal abnormalities were observed in 3% (1 of 29) of galactose-induced *GAL-SRS2* and 0% (0 of 29) glucose-repressed *GAL-srs2*^*K41A*^ diploid cells (Fig 5B). By contrast, 20 of 68 survivors (29%) obtained from galactose-induced *GAL-srs2*^*K41A*^ diploid cells showed abnormal chromosome compositions; both aneuploidy and chromosomal translocations were detected (Fig 5B). Thus, the expression of Srs2^K41A^ in diploids dramatically increases the rates of gross chromosomal rearrangements.

### Srs2 and Mus81–Mms4 are essential for growth of diploid cells

**Fig 6:**
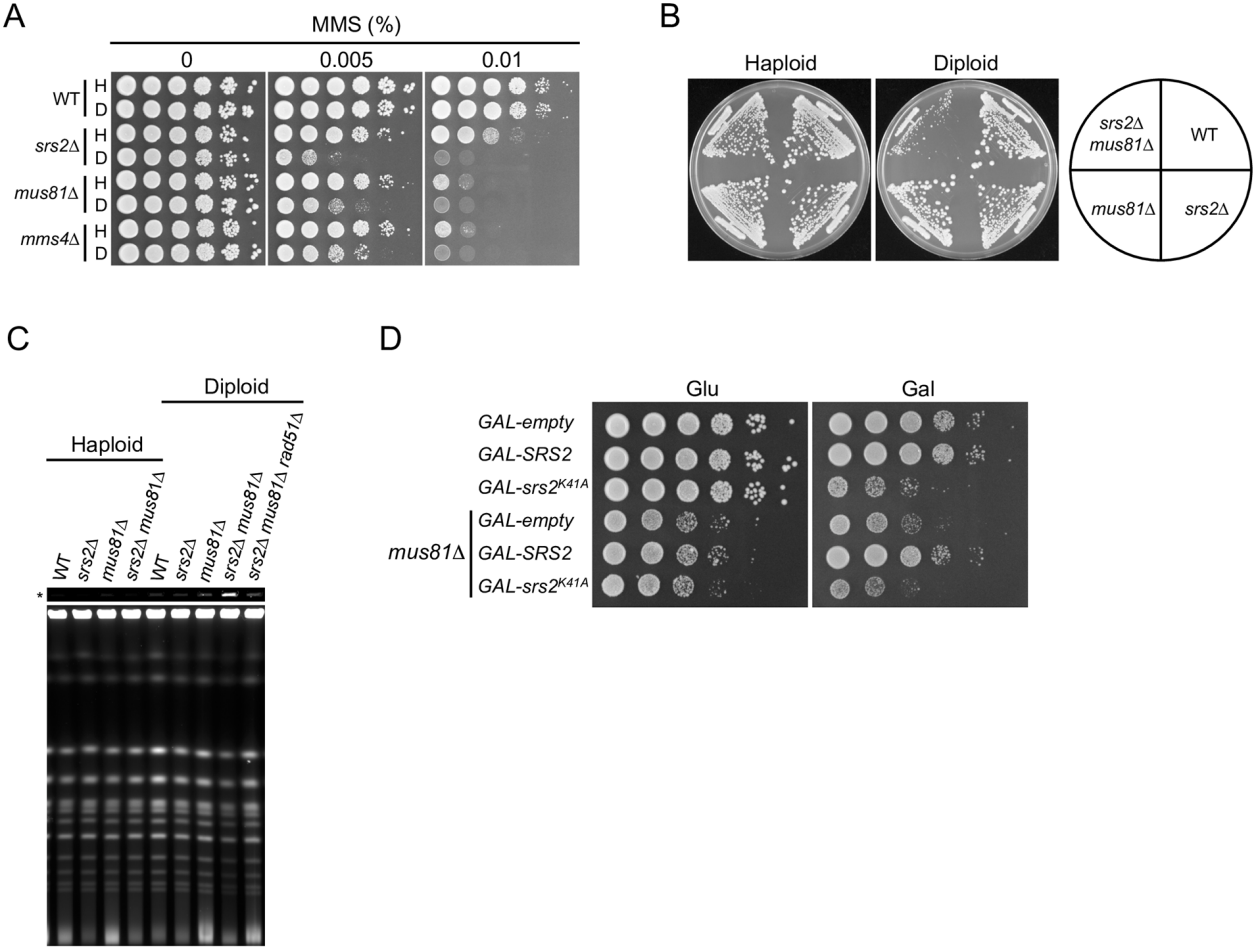
*srs2*Δ *mus81*Δ diploid cells exhibit a severe growth defect. (A) MMS sensitivity of haploid and diploid mutant cells. Cells were grown and spotted on YPD plates containing the indicated concentration of MMS at 10-fold serial dilutions, and incubated at 30°C for 3 days. (B) The indicated haploid and diploid strains were grown on YPD plates at 30°C for 3 days. (C) Accumulation of joint molecules in *srs2Δ mus81*Δ diploid cells, but not in haploid cells. DNA isolated from asynchronous cultures of wild-type (WT), *srs2*Δ, *mus81Δ*, *srs2Δ mus81*Δ, and *srs2Δ mus81Δ rad51Δ* cells was subjected to PFGE and detected by staining with SYBR green. An asterisk (*) indicates well images obtained at low exposure. (D) The effect of Srs2^K41A^ expression on the growth of *srs2*Δ *mus81*Δ diploid cells. The indicated diploid strains grown in YPD were diluted and spotted onto YPD (Glu) and YPR + 0.02% galactose (Gal) plates. These plates were incubated at 30°C for 2 days.

It has been reported that sensitivity to MMS is enhanced in *srs2*Δ diploid cells relative to their haploid counterparts [21]. To gain insight into inter-homolog HR, a genome-wide screen for diploid-specific sensitivity to MMS was conducted using a library (n ≈ 4,800) of viable haploid and diploid deletion mutants, directly testing for a ploidy-specific phenotype in the presence of MMS. The complete results of the screen will be described elsewhere. Three genes were identified that function in the processing of HR intermediates (*SRS2*, *MUS81*, and *MMS4*). Our investigation focused on a subset of HR genes including *SGS1*, *MPH1*, *MUS81*, *MMS4*, *RAD1*, *RAD10*, *YEN1*, *SLX1*, and *SLX4*, which are involved in the processing of D-loops, Holliday junctions, and similar structures [11,17]. We reconfirmed that *mus81*Δ and *mms4*Δ strains were more sensitive to MMS as diploids than as haploids, whereas other HR-deficient diploid strains had similar MMS sensitivity to their haploid counterparts (Fig 6A and S5 Fig). Mus81 interacts with Mms4 to form a structure-specific nuclease, which cleaves a variety of branched structures, including 3' flaps, D-loops, and nicked Holliday junctions [49–51]. These results suggest that Mus81–Mms4 has an important role in the resolution of inter-homolog joint molecules.

The genetic relationship between Srs2 and Mus81–Mms4 was investigated by comparing the growth and viability of haploid and diploid *srs2*Δ and *mus81*Δ single-mutant and double-mutant strains. The *srs2*Δ *mus81*Δ haploid double mutant grew just as well as either single mutant, whereas *srs2*Δ *mus81*Δ diploid cells grew very poorly (Fig 6B). A similar effect was seen in *srs2*Δ *mms4*Δ cells (S6A Fig). Poor growth of *srs2*Δ *mus81*Δ diploids was rescued by expression of plasmid-borne *srs2*^*7AV*^, *srs2*^*3KR*^, or *srs2*^Δ*SIM*^, but not by the plasmid vector alone (S6B Fig, upper panel), which indicated that Srs2 rescued the growth defect of the double-mutant strain in the absence of phosphorylation, sumoylation, or interaction with sumoylated PCNA. However, plasmid-borne *srs2*^Δ*783–998*^, which lacks a Rad51-interaction domain, did not complement the severe growth defect of s*rs2*Δ *mus81*Δ diploid cells (S6B Fig, upper panel), and deletion of *RAD51* or *RAD52* rescued the growth defect (S6C Fig). These results suggest that Mus81–Mms4 and Srs2 have essential roles in inter-homolog HR.

Biochemical and two-hybrid studies have shown that, in addition to Srs2^Δ783–998^, Srs2^Δ875–902^ and Srs2^L844A^ are deficient in Rad51 interaction [52,53]. These results suggest that the amino acid residues of Srs2 that are critical for binding to Rad51 are localized in separate regions within Srs2 residues 783–998. In our study, plasmids were constructed in which *srs2*^*L844A*^, *srs2*^*Δ875–902*^, and *srs2*^*L844A*,*Δ875–902*^ were under the control of the endogenous *SRS2* promoter. Poor growth of *srs2Δ mus81Δ* diploids was rescued by expression of plasmid-borne *SRS2*, *srs2*^*L844A*^, *srs2*^*Δ875–902*^, or *srs2*^*L844A*,*Δ875–902*^, but not *srs2*^*Δ783–998*^ (S6B Fig, lower panel). Similarly, *srs2Δ* diploid cells expressing *srs2*^*K41A*,*L844A*^, *srs2*^*K41A*,*Δ875–902*^, or *srs2*^*K41A*,*L844A*,*Δ875–902*^ from pRS415 were not able to form viable colonies (S6D Fig). These results suggest that Srs2^L844A^ and Srs2^Δ875–902^ retain some ability to interact with Rad51 *in vivo*, consistent with the results of a previous study that the phenotype of *srs2*^*Δ875–902*^ cells is similar to wild-type [52].

### *srs2*Δ *mus81*Δ double mutants accumulate joint molecules in diploid cells

Most *srs2*Δ *mus81*Δ diploids arrested with 4C DNA content and were large-budded cells with a single nucleus, suggesting a significant delay of entry into mitosis (S7 Fig). The simplest explanation for the synergistic growth defect in *srs2*Δ *mus81*Δ diploids is that the double mutants had unresolved inter-homolog joint molecules, which resulted in G_2_/M arrest, as observed in *srs2*^*K41A*^ diploids. PFGE analysis consistently revealed that chromosomal DNA from *srs2*Δ *mus81*Δ diploid cells, but not from their haploid counterparts, formed structures that failed to enter the gel (Fig 6C). Moreover, these DNA accumulations in *srs2Δ mus81Δ* diploid cells were suppressed by the *rad51Δ* mutation (Fig 6C). This ploidy-specific behavior is consistent with our other results, and probably reflects the accumulation of inter-homolog joint molecules.

The phenotypic similarity between *srs2*^*K41A*^ and *srs2Δ mus81Δ* suggested the possible functional interaction between Srs2^K41A^ and Mus81. To address this possibility, *GAL-srs2*^*K41A*^ *mus81Δ*, *GAL-SRS2 mus81Δ*, and *GAL-empty mus81Δ* diploid strains were constructed, and the effect of expressing Srs2^K41A^ or Srs2 in *srs2Δ mus81Δ* diploids in the presence of 0.02% galactose was examined. In a control experiment, expression of wild-type Srs2 complemented the growth defect of the *GAL-SRS2 mus81Δ* mutant (Fig 6D). Notably, whereas Srs2^K41A^ expression aggravated the growth of the *GAL-srs2*^*K41A*^ diploid strain, it had no effect on the growth of the *GAL-srs2*^*K41A*^ *mus81Δ* diploid strain (Fig 6D). These results suggest that the *srs2*^*K41A*^ mutant behaves similarly to the *srs2Δ mus81Δ* double mutant. It should be noted that *srs2*^*K41A*^ was lethal in diploid yeasts, but *srs2Δ mus81Δ* diploid cells were viable, albeit with poor growth, suggesting that the growth defect in *srs2*^*K41A*^ diploids is more toxic than in *srs2Δ mus81Δ* diploids.

## Discussion

### Inter-homolog recombination intermediates accumulate in Srs2^K41A^-expressing diploid cells

Srs2 has a dual function in HR, preventing unscheduled recombination and promoting the SDSA pathway during DSB repair. Our results showed that *srs2*^*K41A*^ diploids, but not haploids, had a severe defect in growth. *GAL-srs2*^*K41A*^ diploid cells showed an elevated number of Rad52 foci and an increase in the rate of gross chromosomal rearrangements, suggesting a high rate of spontaneous HR-associated DNA damage. Indeed, these growth defects were suppressed by inactivation of Rad51 and also by deletion of the Rad51 interaction domain of Srs2^K41A^. These results imply that DSBs are not responsible for the toxic effects of *srs2*^*K41A*^ in diploid yeast, since the repair of DSBs is essential for cell survival and requires functional HR. Indeed, in PFGE analysis, fragmented chromosomes were not detected, but joint molecule accumulations were observed in Srs2^K41A^-expressing diploid cells. To repair ssDNA gaps by HR, homologous sequences located on the same or different chromosomes can serve as templates. Especially, inter-homolog HR occurs only in diploids, whereas inter-sister HR can occur in both haploid and diploid yeasts. Our results suggest, therefore, that the diploid-specific lethality of *srs2*^*K41A*^ is the result of a failure to resolve joint molecules formed during inter-homolog HR.

### Post-translational modification of Srs2^K41A^ is not required for diploid-specific lethality

Srs2 is phosphorylated by Cdk1 and sumoylated in response to DNA damage [33,38]. Cdk1-dependent phosphorylation of Srs2 is required to promote the SDSA pathway for DSB repair. Srs2 sumoylation may have a role in the assembly and/or stabilization of protein complexes involved in DNA repair, although its biological significance remains obscure [40]. In this study, low-abundance Srs2^K41A^ underwent both phosphorylation and sumoylation at multiple sites in haploid and diploid cells, even in the absence of DNA damage. Mutational analysis revealed that sumoylation and phosphorylation of Srs2^K41A^ were largely independent events, which was consistent with the results of a previous study [40]. Moreover, our data demonstrated that Srs2^K41A^ sumoylation and phosphorylation were dispensable for *srs2*^*K41A*^ lethality in diploids. By contrast, the lethality of *srs2*^*K41A*^ in diploids required its interaction with Rad51. These results suggest that the removal of toxic Rad51 filaments by the Srs2 translocase activity may be essential for the viability of diploid cells.

Our results showed that the DNA damage checkpoint, as monitored by Rad53 phosphorylation, was constitutively activated in haploid and diploid cells expressing Srs2^K41A^. A previous study showed that overexpression of wild-type Srs2 in haploid cells activates the DNA damage checkpoint in a manner that requires the Srs2 SIM domain [42]. Similar observations were made in our experiments in *GAL-srs2*^*K41A*^ haploid cells, suggesting that activation of the DNA damage checkpoint in *srs2*^*K41A*^ haploids depends both on DNA replication and the interaction between Srs2 and sumoylated PCNA. By contrast, checkpoint activation and growth inhibition were still observed in *GAL-srs2*^*K41A*,Δ*SIM*^ diploid cells. Thus, *GAL-srs2*^*K41A*,Δ*SIM*^ diploids might accumulate more (or a different type of) DNA lesions than haploid cells of the same genotype, triggering the DNA damage checkpoint. In addition, the *rad51Δ* mutation did not suppress diploid-specific (unrelated to sumoylated PCNA) Rad53 activation of *srs2*^*K41A*,*ΔSIM*^ diploids, suggesting that this checkpoint activation is unlikely to be associated with the lethality of *srs2*^*K41A*^ in diploid cells.

### Mus81 and Srs2 have critical roles in the processing of inter-homolog joint molecules

Inter-homolog recombination intermediates form infrequently during HR in mitotic yeast cells. However, if they form, efficient resolution is required to prevent interference with proper chromosome segregation. Our data suggest that Srs2^K41A^ is recruited to inter-homolog recombination intermediates through its interaction with Rad51, and, further, that Srs2^K41A^ inhibits processing of these intermediates, possibly because it lacks a functional helicase/translocase activity. Thus, a possible explanation for Srs2^K41A^ lethality is that in addition to impeding Srs2-dependent HR, it actively blocks a second repair pathway that resolves inter-homolog joint molecules by other helicases or endonucleases. Srs2 has been shown to physically interact with Mus81–Mms4, and to remove Rad51 from DNA, enabling Mus81–Mms4 to access and cleave DNA [54]. In addition, Srs2 and Mus81 co-localize after DNA damage, although Mus81 is fully proficient in focus formation in the absence of Srs2 [54]. A plausible mechanism for a second repair pathway is the resolution of inter-homolog joint molecules by Mus81–Mms4 endonuclease. In this context, it is notable that each of the *srs2*Δ, *mus81*Δ, and *mms4*Δ mutations resulted in greater sensitivity to MMS in diploids than in haploids, which was not true of *sgs1*Δ mutations. Moreover, *srs2Δ mus81Δ* diploid cells exhibited a severe growth defect and a marked accumulation of joint molecule intermediates, which were also observed in Srs2^K41A^-expressing diploid cells. It remains unclear whether the non-migratory DNA complexes observed during PFGE are direct substrates for Mus81–Mms4. However, our genetic and physical studies showed that the *rad51Δ* mutation could suppress both lethality and joint molecule accumulation in *srs2*^*K41A*^ and *srs2*Δ *mus81*Δ diploids. Moreover, expression of Srs2^K41A^ aggravated the growth of *srs2Δ* diploid cells, whereas it did not affect growth in *srs2*Δ *mus81*Δ diploid cells. Taken together, these findings suggest that the lethality of *srs2*^*K41A*^ and *srs2Δ mus81Δ* diploid cells was likely to be associated with joint molecule accumulation, and that Srs2^K41A^ actively blocks at least in part the Mus81–Mms4 pathway.

These diploid-specific phenotypes of *srs2*^*K41A*^ and *srs2Δ mus81Δ* imply that inter-homolog and inter-sister HR are somewhat mechanistically different in the processing of HR intermediates. Previous studies in haploid yeast reported that the *sgs1*Δ *srs2*Δ and *sgs1*Δ *mus81*Δ double mutants, but not *srs2*Δ *mus81*Δ, are lethal in haploid yeast [55–57]. Sgs1–Top3–Rmi1 (STR) is required to prevent mitotic crossovers by dissolving double Holliday junction structures through the formation of hemicatenanes [8,13,14]. These results indicate that Sgs1 has an important role in dissociating joint molecule intermediates that arise during HR. A possible explanation for the differential results in haploid and diploid yeasts is that some of the inter-homolog joint molecules that accumulate in *srs2*^*K41A*^ and *srs2*Δ *mus81*Δ diploid cells cannot be resolved by the STR complex. Cohesion complexes are recruited to sites of DNA damage independently of DNA replication and have a role in suppressing inter-homolog HR by holding sister chromosomes together [58–60]. We speculate that the STR complex might have limited ability to dissociate inter-homolog joint molecules that contain sister-chromatid cohesin rings because cohesin complexes sterically block the formation of hemicatenanes by restricting the decatenation of topologically linked DNA structures between homologous chromosomes. Alternatively, inter-homolog joint molecules might include specific substrates for Mus81–Mms4, such as a single Holliday junction that cannot be resolved by the STR complex. Indeed, it has been reported that joint molecules formed in the *mus81Δ* mutant contain single Holliday junctions [11].

Our results demonstrate that Srs2 and Mus81–Mms4 participate in essential pathways to prevent the accumulation of toxic inter-homolog joint molecules. In this context, Srs2 may prevent formation of joint molecule structures resulting from inter-homolog HR, whereas Mus81–Mms4 might have a downstream role in promoting their resolution. Unprocessed inter-homolog joint molecules result in chromosome nondisjunction, leading to genetic instability and a high likelihood of cell death. Uncontrolled inter-homolog HR in human cells is associated with genomic instability, such as loss of heterozygosity and gross chromosomal rearrangements, which are hallmarks of cancer cells. Hence, elucidation of the mechanisms controlling inter-homolog HR in diploid yeast could provide new insights into the mechanisms of cancer and aging in humans.

## Materials and Methods

### Strains and plasmids

All yeast strains used in this study are listed in S1 Table (see Supporting Information). All double mutants and triple mutants were constructed by standard genetic methods. The details of strains and plasmids produced for and used in this study are presented in S1 File (see Supporting Information).

### Media and growth conditions

Cells were grown in yeast extract–peptone–dextrose medium containing 0.01% adenine sulfate (YPD) at 30°C. Cells transformed with pRS415 derivatives were selected on Synthetic Complete (SC)+Glucose medium lacking leucine (SC+Glu-Leu). For Srs2 expression from the *AUR1* locus, cells grown exponentially in YPD or YP-2% raffinose (YPR) medium were further incubated at 30°C for various times in YPR medium containing galactose. In a mating assay to produce diploid cells, the resulting diploid cells were selected on SC+Glu medium lacking both histidine and leucine. Disome cells transformed with pRS415 derivatives were selected on SC+Glu medium lacking both leucine and histidine and containing 300 μg/mL G418 (Sigma-Aldrich). Cells resistant to both canavanine and 5-fluoroorotic acid (5-FOA) were selected on SC+Glu medium lacking arginine and containing 60 μg/mL canavanine and 1 mg/mL 5-FOA. For Srs2 expression from p415GAL1 derivatives, cells grown in SC+Glu-Leu medium were washed with water, and the cells (1×10^7^ cells/mL) were further incubated at 30°C for 6 h in SC-Leu medium containing 2% raffinose and 0.2% galactose. For Srs2 expression from the *AUR1* locus of disome strains, cells grown in SC+Glu-His+G418 medium containing 0.05 μg/mL aureobasidin A were washed with water, and the cells (2×10^6^ cells/mL) were further incubated at 30°C for 3 h in SC-His+G418 medium containing 2% raffinose in place of glucose and then transferred to 0.5% galactose-containing medium.

### Preparation of yeast extracts and western blotting

Protein extracts were prepared from 1×10^8^ cells using the trichloroacetic acid (TCA) method, as described previously [61]. Proteins were separated by SDS-PAGE, transferred to PVDF membrane, and probed with anti-Srs2 or anti-Rad53 polyclonal antibodies (Santa Cruz).

### PFGE analysis

Yeast chromosomes were separated with CHEF-Mapper XA (Bio-Rad) in 0.8% agarose with 0.5×TBE buffer and stained using ethidium bromide or SYBR Green I (Life Technologies). Gel images were acquired with an LAS4000 mini system (GE Healthcare). The intensity of chromosome bands was quantified using Image J software (NIH). For samples digested with *Not*I and RuvC, the plugs prepared for PFGE were washed twice more with H buffer (Takara) containing 0.01% Triton X-100 and then washed twice with the same buffer containing 1.3 mM phenylmethylsulfonyl fluoride (PMSF). The plugs were treated with 300 units/mL *Not*I at 37°C for 16 h in the same buffer. The *Not*I treated plugs were washed twice with a buffer containing 20 mM Tris-HCl (pH7.5), 10 mM Mg(OAc)_2_, and 1 mM DTT, and then washed twice with the same buffer containing 1.3 mM PMSF. The plugs were further incubated at 37°C for 16 h in the same buffer containing 8 μg/mL RuvC. After incubation, the plugs were treated with proteinase K and washed twice with 0.5×TBE for PFGE analysis. Southern blotting was performed essentially as described previously [62]. Chromosomes were transferred to a charged nylon membrane (Hybond-N+; GE Healthcare) and hybridized with alkaline phosphatase-labeled probes, which were prepared from PCR products (chromosome IV; 463,680–463,707). After hybridization, the membrane was treated with CDP-Star (GE Healthcare), and chromosomes were detected with the LAS4000 mini imaging system.

### Chromosome-loss frequency

The frequency of loss of a pair of chromosome V homologs marked with *CAN1* on the right arm and *URA3* on the left arm was determined. Briefly, cells were grown in SC+Glu medium lacking histidine and uracil, and further incubated at 30°C for 3 h in YPR medium. After incubation, galactose (0.02%) was added to the cultures, followed by incubation at 30°C for 8 h. Cells from each culture were washed and spread onto plates at an appropriate dilution to determine the total cell number (on YPD plates) and the cell number with allelic loss of chromosome V (on SC+Glu plates containing canavanine and 5-FOA). Colonies arising on YPD and SC+Glu plates containing canavanine and 5-FOA were counted after 3 or 4 days of growth at 30°C. The chromosome-loss frequency was determined from the number of colonies with both Can^R^ and 5-FOA^R^ per mL divided by the number of viable cells per mL, and the average from three independent experiments was calculated. p values were calculated using a Student’s two-tailed t test.

### Other methods

Fluorescence-activated cell sorting (FACS) analysis, microscopy, and spot assays for MMS sensitivity were performed as described previously [63].

## Supporting Information

S1 Fig Characterization of the *GAL-SRS2* and *GAL-srs2*^*K41A*^ strains. (A) Cells grown in YPD medium for 8 h were stained with DAPI to evaluate nuclear and cellular morphology under a microscope. The results show the averages of three independent measurements. Error bars indicate the standard error for each data point. (B) *GAL-SRS2* diploid cells were grown at 30°C in YPR medium containing various concentrations of galactose, and cells were harvested at 6 h. Protein extracts were prepared and separated by 6% SDS-PAGE, followed by western blotting with anti-Srs2 antibodies. (C) Wild-type, *srs2*Δ, and *GAL-SRS2* diploid cells grown in YPD medium were diluted and spotted onto YPD plates and YPR plates containing 0.02% or 0.2% galactose. These plates were incubated at 30°C for 2 days. (D) The indicated diploid strains grown in YPD medium were diluted and spotted onto YPD plates and YPR plates containing 0.02% or 0.05% galactose. These plates were incubated at 30°C for 2 days.
(PDF)

S2 Fig Analysis of GFP-fused alpha-tubulin foci in *GAL-srs2*^*K41A*^ diploid cells. *GAL-srs2*^*K41A*^ diploid cells were grown at 30°C for 8 h in YPD or YPR + 0.02% galactose medium. Cells were collected, stained with DAPI, and examined by fluorescence microscopy. Representative images of Tub1-GFP foci and DAPI staining are shown.
(PDF)

S3 Fig PFGE analysis and Rad53 phosphorylation of *GAL-srs2*^*K41A*^ diploid cells. (A) *GAL-srs2*^*K41A*^ diploid cells grown in YPR + 0.02% galactose medium were collected at the indicated time points. Chromosomal DNA was separated by PFGE and detected by staining with SYBR green. “*well” indicates images taken at low exposure. (B) The indicated diploid strains were grown in YPD medium. Cells were transferred to YPR + 0.02% galactose medium to induce Srs2 expression and then cultured at 30°C for 6 h. Protein extracts were prepared and separated by 6% SDS-PAGE, followed by western blotting with an anti-Rad53 antibody. (C) *GAL-srs2*^*K41A*^ diploid cells were grown at 30°C for 4 h in YPR or YPR + 0.02% galactose. Chromosomal DNA was isolated in agarose-gel blocks, digested with RuvC at 37°C for 16 h, and subjected to PFGE as described above. “*well” indicates images taken at low exposure.
(PDF)

S4 Fig Analysis of *GAL-srs2*^*K41A*^ disome IV cells. (A) *srs2*Δ and *srs2Δ rad51Δ* haploid cells or *srs2*Δ and *srs2Δ rad51Δ* disome IV cells were transformed with pRS415 vector derivatives bearing *SRS2* or *srs2*^*K41A*^, and the plates were incubated at 30°C for 3 days on plates containing SC+Glu medium lacking leucine and histidine and containing G418. (B) The indicated haploid and disome IV strains grown in SC+Glu-His+G418 were diluted and spotted onto SC-His+G418 containing 2% glucose or 2% raffinose + 0.5% galactose. These plates were incubated at 30°C for 3 days. (C) The *GAL-empty* disome IV and the *GAL-SRS2* disome IV strains were transferred to SC-His+G418 containing 2% raffinose + 0.5% galactose, and incubated for the indicated times. Chromosomal DNA was separated by PFGE and stained with SYBR green. “M” indicates haploid DNA as a size marker. The band intensities of chromosomes IV (circle), VII+XV (square), and XI (triangle) detected by staining the gel were quantified and are shown relative to 100% at time 0.
(PDF)

S5 Fig Screening for diploid-specific methyl methanesulfonate (MMS)-sensitive mutants. The indicated haploid deletion mutants (H) and their diploid counterparts (D) grown in YPD medium were diluted and spotted onto YPD plates containing MMS (0%, 0.005%, 0.01%, and 0.02%). These plates were incubated at 30°C for 3 days.
(PDF)

S6 Fig Analysis of *srs2Δ mms4Δ* and *srs2Δ mus81Δ* diploid cells. (A) The indicated haploid and diploid strains were grown on YPD plates at 30°C for 3 days. (B) *srs2*Δ *mus81*Δ diploid cells carrying the indicated plasmids were streaked onto SC+Glu-Leu plates. The plates were incubated at 30°C for 3 days. (C) The *rad51*Δ or *rad52*Δ mutations suppress the severe growth defect of *srs2*Δ *mus81*Δ diploid cells. Cells were streaked onto YPD plates, and the plates were incubated at 30°C for 3days. (D) The *srs2*Δ haploid or diploid strains were transformed with pRS415 derivatives carrying *SRS2*, *srs2*^*K41A*^, *srs2*^*K41A*,*L844A*^, *srs2*^*K41A*,*Δ875–902*^, and *srs2*^*K41A*,*L844A*,*Δ875–902*^, and the plates were incubated at 30°C for 3 days.
(PDF)

S7 Fig Fluorescence-activated cell sorting (FACS) analysis of *srs2Δ mus81Δ* diploid cells. Asynchronous diploid cells were grown at 30°C in YPD medium, and samples were collected. DNA content was measured by FACS. The same samples were stained with DAPI to visualize the DNA, and then observed by microscopy.
(PDF)

S1 Table *S*. *cerevisiae* strains used in this study. (DOCX)

S1 File Construction of strains and plasmids. (DOCX)
