# Supplementary figures and images for: Srs2 and Mus81–Mms4 Prevent Accumulation of Toxic Inter-Homolog Recombination Intermediates
Source: PLoS Genet. 2016 Jul 7;12(7):e1006136. doi: 10.1371/journal.pgen.1006136 (PMC4936719; doi:10.1371/journal.pgen.1006136)

S1 Fig. Characterization of the *GAL-SRS2* and *GAL-srs2<sup>K41A</sup>* strains.

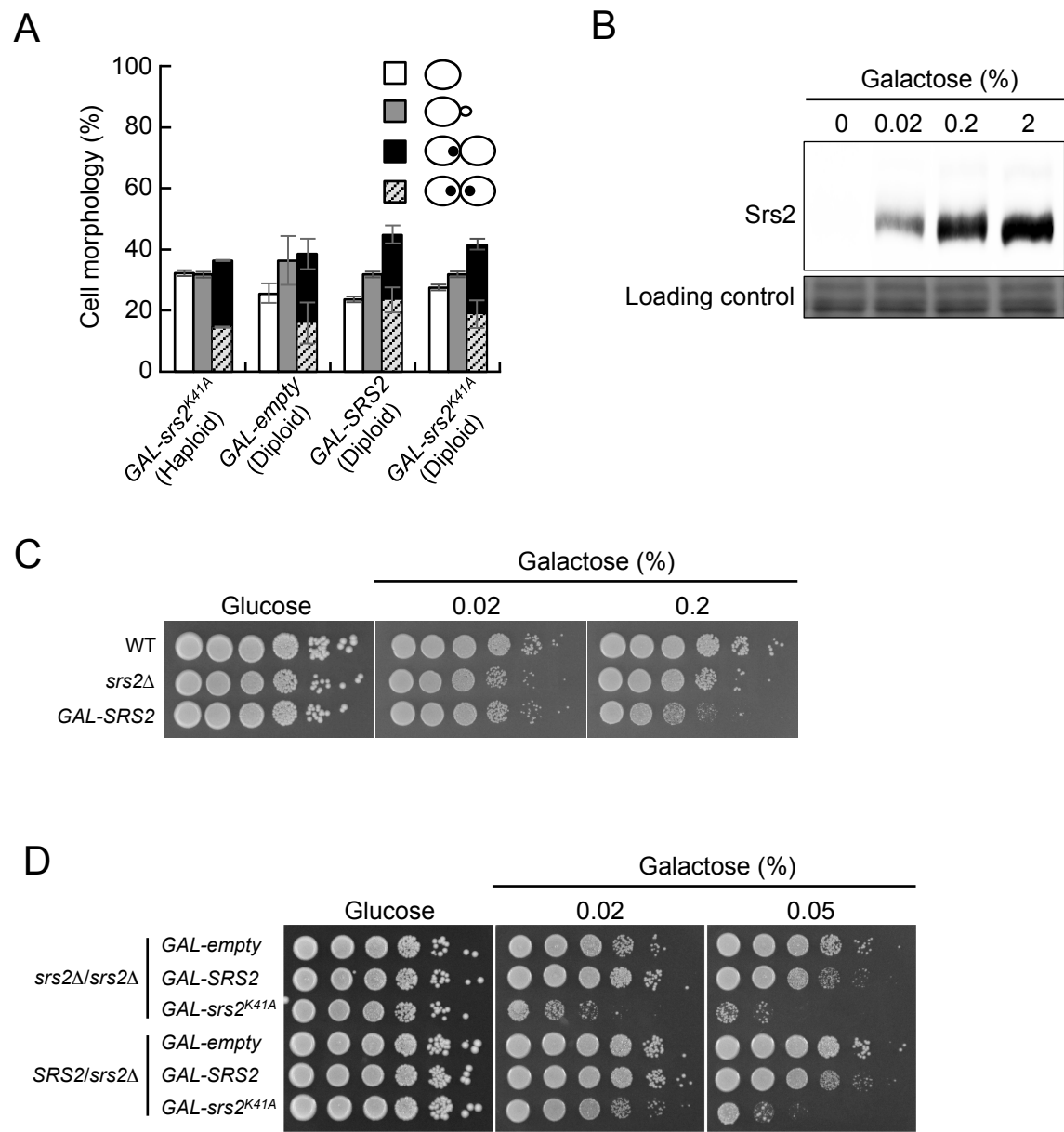

Supplement: S1 Fig — (A) Cells grown in YPD medium for 8 h were stained with DAPI to evaluate nuclear and cellular morphology under a microscope. The results show the averages of three independent measurements. Error bars indicate the standard error for each data point. (B) GAL-SRS2 diploid cells were grown at 30°C in YPR medium containing various concentrations of galactose, and cells were harvested at 6 h. Protein extracts were prepared and separated by 6% SDS-PAGE, followed by western blotting with anti-Srs2 antibodies. (C) Wild-type, srs2Δ, and GAL-SRS2 diploid cells grown in YPD medium were diluted and spotted onto YPD plates and YPR plates containing 0.02% or 0.2% galactose. These plates were incubated at 30°C for 2 days. (D) The indicated diploid strains grown in YPD medium were diluted and spotted onto YPD plates and YPR plates containing 0.02% or 0.05% galactose. These plates were incubated at 30°C for 2 days. (PDF) [file pgen.1006136.s001.pdf]

**S2 Fig. Analysis of GFP-fused alpha-tubulin foci in *GAL-srs2<sup>K41A</sup>* diploid cells.**

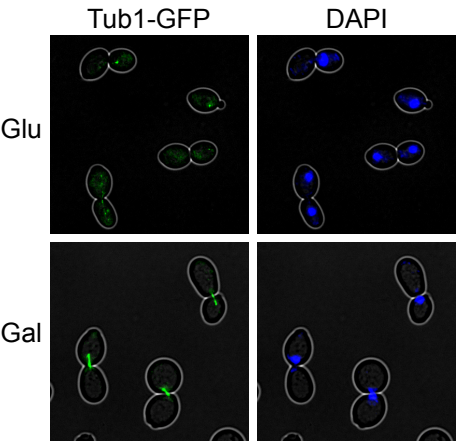

Supplement: S2 Fig — GAL-srs2K41A diploid cells were grown at 30°C for 8 h in YPD or YPR + 0.02% galactose medium. Cells were collected, stained with DAPI, and examined by fluorescence microscopy. Representative images of Tub1-GFP foci and DAPI staining are shown. (PDF) [file pgen.1006136.s002.pdf]

S3 Fig. PFGE analysis and Rad53 phosphorylation of *GAL-srs2<sup>K41A</sup>* diploid cells.

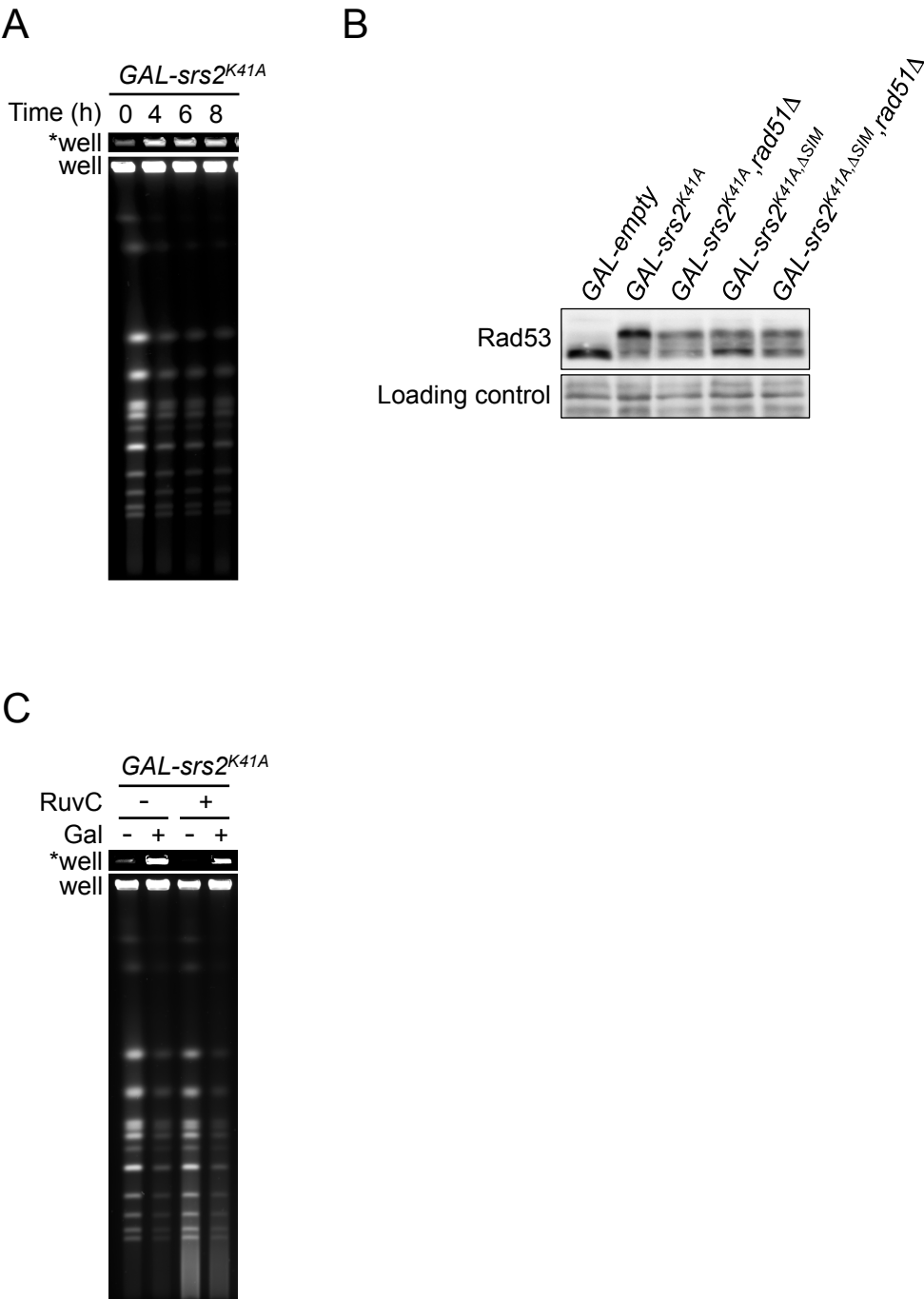

Supplement: S3 Fig — (A) GAL-srs2K41A diploid cells grown in YPR + 0.02% galactose medium were collected at the indicated time points. Chromosomal DNA was separated by PFGE and detected by staining with SYBR green. “*well” indicates images taken at low exposure. (B) The indicated diploid strains were grown in YPD medium. Cells were transferred to YPR + 0.02% galactose medium to induce Srs2 expression and then cultured at 30°C for 6 h. Protein extracts were prepared and separated by 6% SDS-PAGE, followed by western blotting with an anti-Rad53 antibody. (C) GAL-srs2K41A diploid cells were grown at 30°C for 4 h in YPR or YPR + 0.02% galactose. Chromosomal DNA was isolated in agarose-gel blocks, digested with RuvC at 37°C for 16 h, and subjected to PFGE as described above. “*well” indicates images taken at low exposure. (PDF) [file pgen.1006136.s003.pdf]

S4 Fig. Analysis of *GAL-srs2<sup>K41A</sup>* disome IV cells.

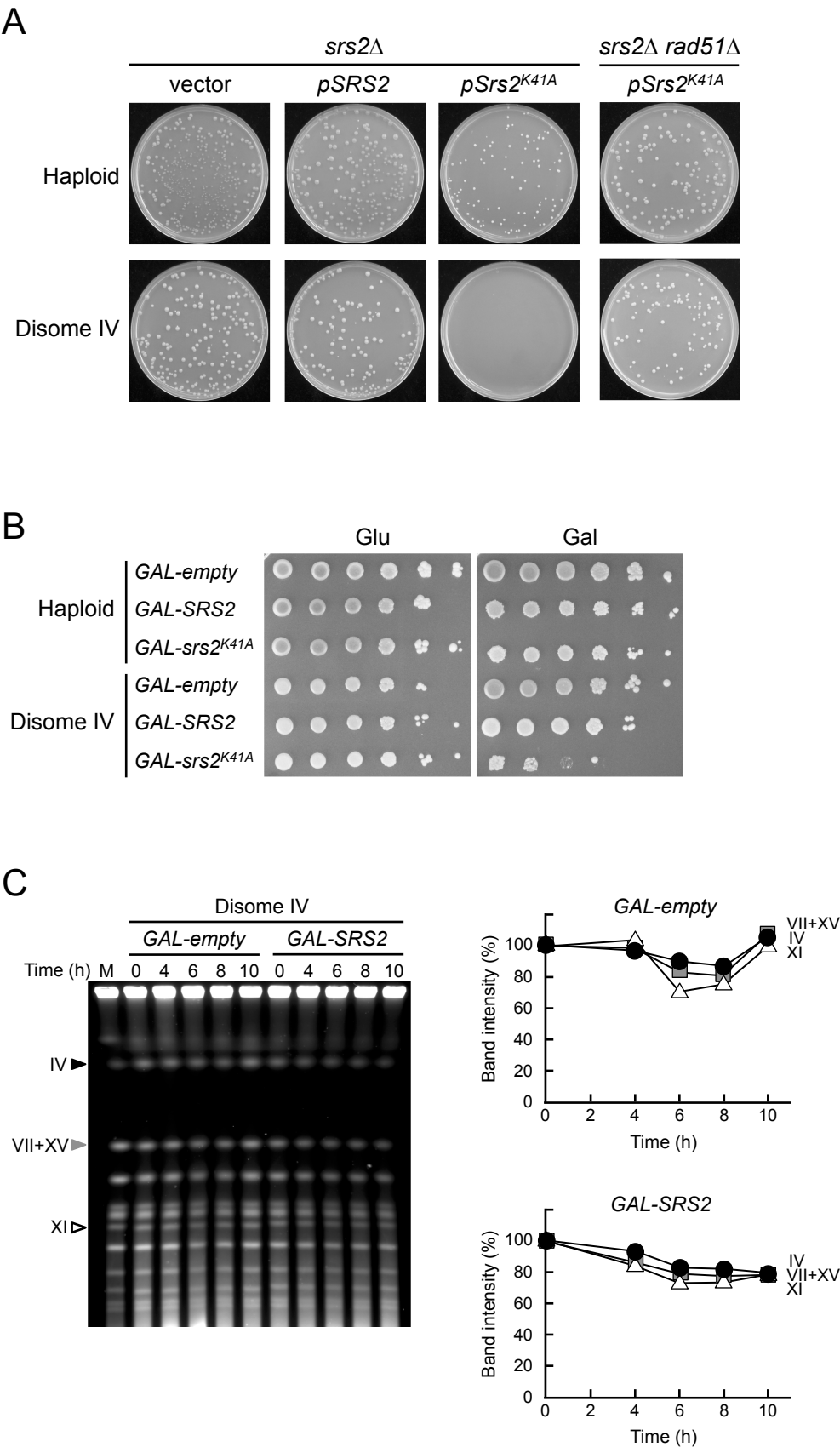

Supplement: S4 Fig — (A) srs2Δ and srs2Δ rad51Δ haploid cells or srs2Δ and srs2Δ rad51Δ disome IV cells were transformed with pRS415 vector derivatives bearing SRS2 or srs2K41A, and the plates were incubated at 30°C for 3 days on plates containing SC+Glu medium lacking leucine and histidine and containing G418. (B) The indicated haploid and disome IV strains grown in SC+Glu-His+G418 were diluted and spotted onto SC-His+G418 containing 2% glucose or 2% raffinose + 0.5% galactose. These plates were incubated at 30°C for 3 days. (C) The GAL-empty disome IV and the GAL-SRS2 disome IV strains were transferred to SC-His+G418 containing 2% raffinose + 0.5% galactose, and incubated for the indicated times. Chromosomal DNA was separated by PFGE and stained with SYBR green. “M” indicates haploid DNA as a size marker. The band intensities of chromosomes IV (circle), VII+XV (square), and XI (triangle) detected by staining the gel were quantified and are shown relative to 100% at time 0. (PDF) [file pgen.1006136.s004.pdf]

S5 Fig. Screening for diploid-specific MMS sensitive mutants.

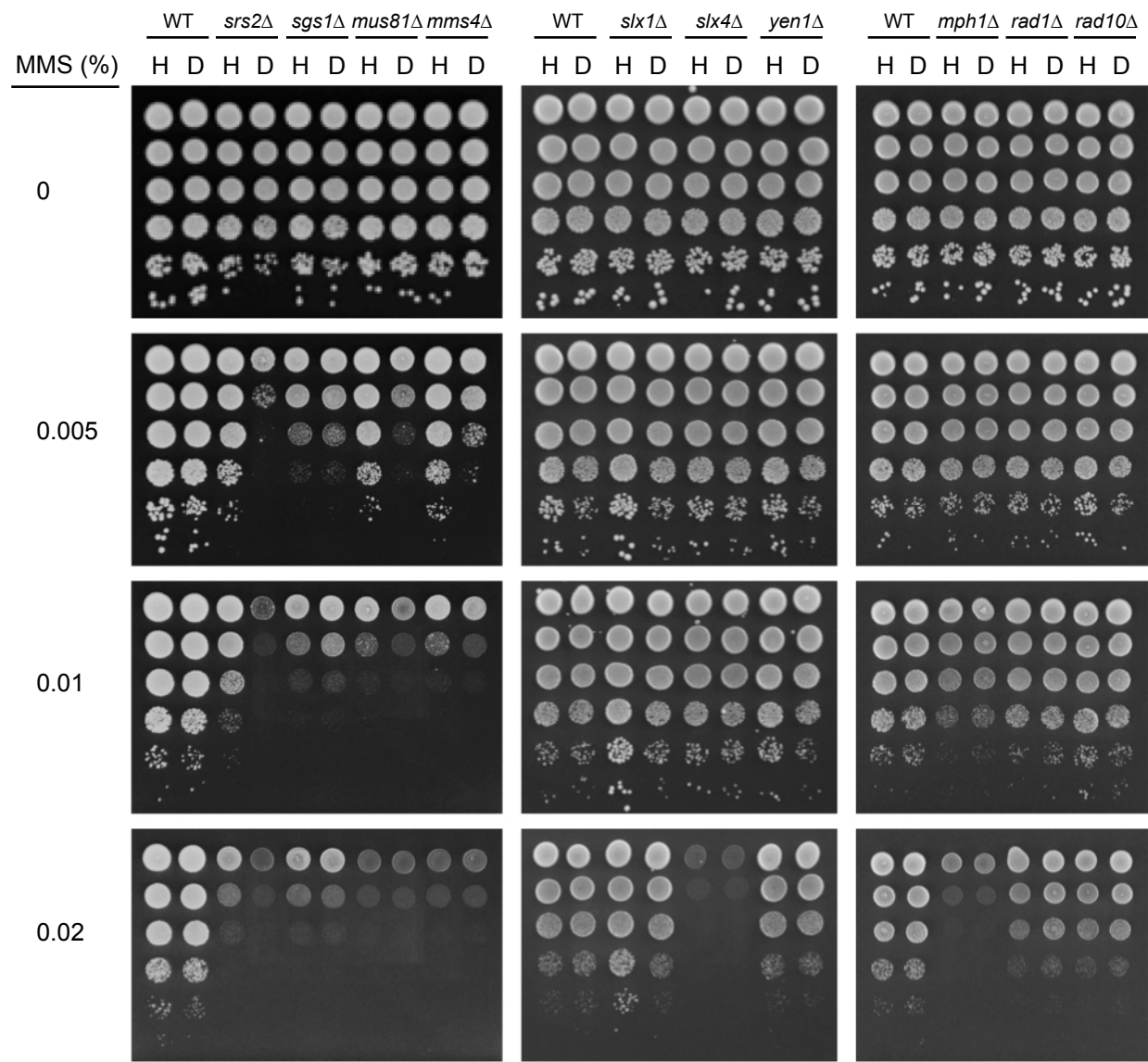

Supplement: S5 Fig — The indicated haploid deletion mutants (H) and their diploid counterparts (D) grown in YPD medium were diluted and spotted onto YPD plates containing MMS (0%, 0.005%, 0.01%, and 0.02%). These plates were incubated at 30°C for 3 days. (PDF) [file pgen.1006136.s005.pdf]

S6 Fig. Analysis of *srs2Δ mms4Δ* and *srs2Δ mus81Δ* diploid cells

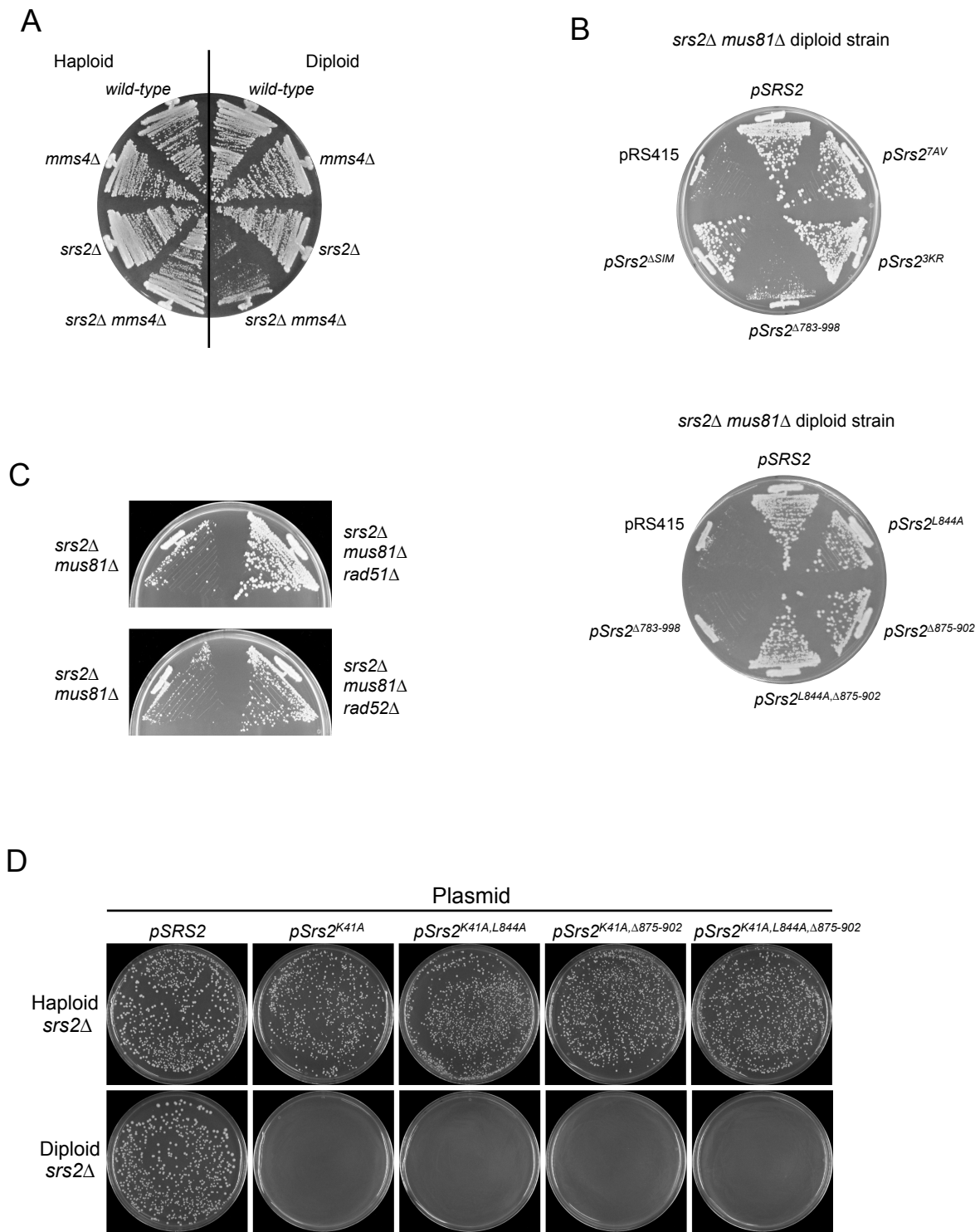

Supplement: S6 Fig — (A) The indicated haploid and diploid strains were grown on YPD plates at 30°C for 3 days. (B) srs2Δ mus81Δ diploid cells carrying the indicated plasmids were streaked onto SC+Glu-Leu plates. The plates were incubated at 30°C for 3 days. (C) The rad51Δ or rad52Δ mutations suppress the severe growth defect of srs2Δ mus81Δ diploid cells. Cells were streaked onto YPD plates, and the plates were incubated at 30°C for 3days. (D) The srs2Δ haploid or diploid strains were transformed with pRS415 derivatives carrying SRS2, srs2K41A, srs2K41A,L844A, srs2K41A,Δ875–902, and srs2K41A,L844A,Δ875–902, and the plates were incubated at 30°C for 3 days. (PDF) [file pgen.1006136.s006.pdf]

S7 Fig. FACS analysis of *srs2Δ mus81Δ* diploid cells.

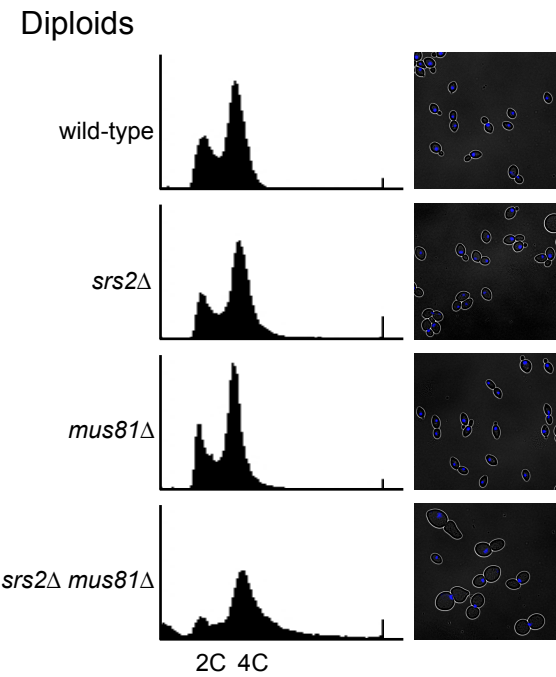

Supplement: S7 Fig — Asynchronous diploid cells were grown at 30°C in YPD medium, and samples were collected. DNA content was measured by FACS. The same samples were stained with DAPI to visualize the DNA, and then observed by microscopy. (PDF) [file pgen.1006136.s007.pdf]
