# Supplementary material for: Srs2 and Mus81–Mms4 Prevent Accumulation of Toxic Inter-Homolog Recombination Intermediates
Source: PLoS Genet. 2016 Jul 7;12(7):e1006136. doi: 10.1371/journal.pgen.1006136 (PMC4936719; doi:10.1371/journal.pgen.1006136)
Supplement: S1 Table — (DOCX) [file pgen.1006136.s008.docx]

**S1 Table. *S. cerevisiae* strains used in this study.**

| Strain | Genotype | Source |
| --- | --- | --- |
| BY4741 | *MAT*a *leu2*∆*0* *ura3*∆*0* *his3*∆*1* *met15*∆*0* | ATCC* |
| BY4742 | *MAT*α *leu2*∆*0* *ura3*∆*0* *his3*∆*1* *lys2*∆*0* | ATCC |
| BY4743 | *MAT*a/α BY4741/BY4742 | ATCC |
| KK0001 | BY4741 *srs2*∆::*KanMX4* | [[1](#_ENREF_1)] |
| KK0002 | BY4743 *srs2*∆::*KanMX4*/*srs2*∆::*KanMX4* | [[1](#_ENREF_1)] |
| KK0003 | BY4741 *SRS2*::*HIS3* | This study |
| KK0004 | BY4741 *srs2^K41A^*::*HIS3* | This study |
| TH602 | BY4741 *srs2*∆::*HIS3* | [[2](#_ENREF_2)] |
| KK0005 | BY4742 *SRS2*::*LEU2* | This study |
| KK0006 | BY4742 *srs2^K41A^*::*LEU2* | This study |
| KK0007 | KK0001 *aur1C* | This study |
| KK0008 | KK0001 *aur1C*::*GAL1p*-*SRS2* | This study |
| KK0009 | KK0001 *aur1C*::*GAL1p*-*srs2^K41A^* | This study |
| KK0010 | KK0002 *aur1C* | This study |
| KK0011 | KK0002 *aur1C*::*GAL1p*-*SRS2* | This study |
| KK0012 | KK0002 *aur1C*::*GAL1p*-*srs2^K41A^* | This study |
| KK0013 | BY4743 *srs2*∆::*HIS3*/*srs2*∆::*HIS3* *rad51*∆::*URA3*/*rad51*∆::*URA3* | This study |
| KK0014 | KK0007 *rad51*∆::*HIS3* | This study |
| KK0015 | KK0008 *rad51*∆::*HIS3* | This study |
| KK0016 | KK0009 *rad51*∆::*HIS3* | This study |
| KK0017 | KK0010 *rad51*∆::*HIS3*/*rad51*∆::*HIS3* | This study |
| KK0018 | KK0011 *rad51*∆::*HIS3*/*rad51*∆::*HIS3* | This study |
| KK0019 | KK0012 *rad51*∆::*HIS3*/*rad51*∆::*HIS3* | This study |
| KK0020 | KK0001 *aur1C*::*GAL1p*-*srs2*^∆^*^SIM^* | This study |
| KK0021 | KK0001 *aur1C*::*GAL1p*-*srs2^K41A,^*^∆^*^SIM^* | This study |
| KK0022 | KK0002 *aur1C*::*GAL1p*-*srs2*^∆^*^SIM^* | This study |
| KK0023 | KK0002 *aur1C*::*GAL1p*-*srs2^K41A,^*^∆^*^SIM^* | This study |
| KK0024 | KK0009 *RAD52-GFP*::*HIS3* | This study |
| KK0025 | KK0010 *RAD52-GFP*::*HIS3*/*RAD52-GFP*::*HIS3* | This study |
| KK0026 | KK0011 *RAD52-GFP*::*HIS3*/*RAD52-GFP*::*HIS3* | This study |
| KK0027 | KK0012 *RAD52-GFP*::*HIS3*/*RAD52-GFP*::*HIS3* | This study |
| KK0028 | KK0010 *HOM3*/*hom3*∆::*URA3* *can1*∆::*HIS3*/*CAN1* | This study |
| KK0029 | KK0011 *HOM3*/*hom3*∆::*URA3* *can1*∆::*HIS3*/*CAN1* | This study |
| KK0030 | KK0012 *HOM3*/*hom3*∆::*URA3* *can1*∆::*HIS3*/*CAN1* | This study |
| KK0031 | BY4741 *mus81*∆::*KanMX4* | [[1](#_ENREF_1)] |
| KK0032 | BY4741 *mus81*∆::*HIS3* | This study |
| KK0033 | BY4743 *mus81*∆::*KanMX4*/*mus81*∆::*KanMX4* | [[1](#_ENREF_1)] |
| KK0034 | Y4743 *mus81*∆::*HIS3*/*mus81*∆::*HIS3* | This study |
| KK0035 | BY4741 *mms4*∆::*KanMX4* | [[1](#_ENREF_1)] |
| KK0036 | BY4743 *mms4*∆::*KanMX4*/*mms4*∆::*KanMX4* | [[1](#_ENREF_1)] |
| KK0037 | KK0001 *mus81*∆::*HIS3* | This study |
| KK0038 | KK0002 *mus81*∆::*HIS3*/*mus81*∆::*HIS3* | This study |
| KK0039 | KK0038 *rad51*∆::*URA3*/*rad51*∆::*URA3* | This study |
| KK0040 | KK0038 *rad52*∆::*URA3*/*rad52*∆::*URA3* | This study |
| A2587 | *MAT*a *ade2-1* *leu2-3* *ura3* *trp1-1* *his3-11,-15* *can1-100* *GAL* *psi+* | [[3](#_ENREF_3)] |
| A11311 | A2587 *ade1*::*HIS3* *lys2*::*KanMX6* | [[3](#_ENREF_3)] |
| KK0041 | A11311 *srs2*∆::*URA3* | This study |
| KK0042 | A11311 *srs2*∆::*URA3* *aur1C* | This study |
| KK0043 | A11311 *srs2*∆::*URA3* *aur1C*::*GAL1p*-*SRS2* | This study |
| KK0044 | A11311 *srs2*∆::*URA3* *aur1C*::*GAL1p*-*srs2^K41A^* | This study |
| A12687 | A2587 *trp1*::*HIS3*/*trp1*::*KanMX6* (disome IV) | [[3](#_ENREF_3)] |
| KK0045 | A12687 *srs2*∆::*URA3* | This study |
| KK0046 | A12687 *srs2*∆::*URA3* *aur1C* | This study |
| KK0047 | A12687 *srs2*∆::*URA3* *aur1C*::*GAL1p*-*SRS2* | This study |
| KK0048 | A12687 *srs2*∆::*URA3* *aur1C*::*GAL1p*-*srs2^K41A^* | This study |
| KK0049 | KK0041 *rad51*∆::*TRP1* | This study |
| KK0050 | KK0045 *rad51*∆::*TRP1* | This study |
| KK0051 | BY4741 *sgs1*∆::*KanMX4* | [[1](#_ENREF_1)] |
| KK0052 | BY4743 *sgs1*∆::*KanMX4*/*sgs1*∆::*KanMX4* | [[1](#_ENREF_1)] |
| KK0053 | BY4741 *slx1*∆::*KanMX4* | [[1](#_ENREF_1)] |
| KK0054 | BY4743 *slx1*∆::*KanMX4*/*slx1*∆::*KanMX4* | [[1](#_ENREF_1)] |
| KK0055 | BY4741 *slx4*∆::*KanMX4* | [[1](#_ENREF_1)] |
| KK0056 | BY4743 *slx4*∆::*KanMX4*/*slx4*∆::*KanMX4* | [[1](#_ENREF_1)] |
| KK0057 | BY4741 *yen1*∆::*KanMX4* | [[1](#_ENREF_1)] |
| KK0058 | BY4743 *yen1*∆::*KanMX4*/*yen1*∆::*KanMX4* | [[1](#_ENREF_1)] |
| KK0059 | BY4741 *mph1*∆::*KanMX4* | [[1](#_ENREF_1)] |
| KK0060 | BY4743 *mph1*∆::*KanMX4*/*mph1*∆::*KanMX4* | [[1](#_ENREF_1)] |
| KK0061 | BY4741 *rad1*∆::*KanMX4* | [[1](#_ENREF_1)] |
| KK0062 | BY4743 *rad1*∆::*KanMX4*/*rad1*∆::*KanMX4* | [[1](#_ENREF_1)] |
| KK0063 | BY4741 *rad10*∆::*KanMX4* | [[1](#_ENREF_1)] |
| KK0064 | BY4743 *rad10*∆::*KanMX4*/*rad10*∆::*KanMX4* | [[1](#_ENREF_1)] |
| KK0065 | KK0012 *TUB1*-*GFP*::*HIS3*/*TUB1* | This study |
| KK0066 | KK0035 *srs2*∆::*URA3* | This study |
| KK0067 | KK0036 *srs2*∆::*URA3*/*srs2*∆::*HIS3* | This study |
| KK0068 | BY4743 *SRS2*/*srs2*∆::*KanMX4 aur1C* | This study |
| KK0069 | BY4743 *SRS2*/*srs2*∆::*KanMX4 aur1C*::*GAL1p*-*SRS2* | This study |
| KK0070 | BY4743 *SRS2*/*srs2*∆::*KanMX4 aur1C*::*GAL1p*-*srs2^K41A^* | This study |
| KK0071 | KK0010 *mus81*∆::*HIS3*/*mus81*∆::*HIS3* | This study |
| KK0072 | KK0011 *mus81*∆::*HIS3*/*mus81*∆::*HIS3* | This study |
| KK0073 | KK0012 *mus81*∆::*HIS3*/*mus81*∆::*HIS3* | This study |
| KK0074 | KK0023 *rad51*∆::*HIS3*/*rad51*∆::*HIS3* | This study |

* American type culture collection

**References**

1. Winzeler EA, Shoemaker DD, Astromoff A, Liang H, Anderson K, et al. (1999) Functional characterization of the S. cerevisiae genome by gene deletion and parallel analysis. Science 285: 901-906.

2. Hishida T, Hirade Y, Haruta N, Kubota Y, Iwasaki H (2010) Srs2 plays a critical role in reversible G2 arrest upon chronic and low doses of UV irradiation via two distinct homologous recombination-dependent mechanisms in postreplication repair-deficient cells. Mol Cell Biol 30: 4840-4850.

3. Sheltzer JM, Blank HM, Pfau SJ, Tange Y, George BM, et al. (2011) Aneuploidy drives genomic instability in yeast. Science 333: 1026-1030.
