# Supplementary material for: Srs2 and Mus81–Mms4 Prevent Accumulation of Toxic Inter-Homolog Recombination Intermediates
Source: PLoS Genet. 2016 Jul 7;12(7):e1006136. doi: 10.1371/journal.pgen.1006136 (PMC4936719; doi:10.1371/journal.pgen.1006136)
Supplement: S1 File — (DOCX) [file pgen.1006136.s009.docx]

**S1 File. Construction of strains and plasmids.**

The plasmid pUC19-*SRS2* was constructed by cloning a *Bam*HI fragment (3.9 kb) containing the endogenous *SRS2* promoter and the coding region of wild-type *SRS2* into pUC19. Plasmid constructs for *srs2^K41A^*, *srs2^3KR^*, and *srs2*^∆^*^SIM^* were generated from pUC19-*SRS2* by site-directed mutagenesis, resulting in pUC19-*srs2^K41A^*, pUC19-*srs2^3KR^*, and pUC19-*srs2*^∆^*^SIM^*, respectively. A *Bam*HI fragment containing the *srs2^7AV^* allele was amplified by PCR using genomic DNA [[1](#_ENREF_1)], and cloned into pUC19, resulting in pUC19-*srs2^7AV^*. In *srs2^K41A^*, the invariant lysine in the Walker A motif is replaced with alanine. In *srs2^3KR^*, the three lysines in sumoylation motifs, K1081, K1089, and K1142 are mutated to arginine [[2](#_ENREF_2)]. *srs2*^∆^*^SIM^* lacks the SUMO-interacting motif (SIM) consisting of six C-terminal amino acid residues, which mediates interaction with sumoylated PCNA [[3](#_ENREF_3)]. *srs2^7AV^* contains alanine/valine substitutions in the seven serine/threonine Cdk1 consensus sites, which abolishes DNA damage-induced phosphorylation of Srs2 [[4](#_ENREF_4),[5](#_ENREF_5)]. Plasmid constructs for *srs2^K41A^*^,^*^7AV^* or *srs2^K41A,^*^∆^*^SIM^* were obtained from pUC19-*srs2^7AV^* or pUC19-*srs2*^∆^*^SIM^* by the same method as above, resulting in pUC19-*srs2^K41A^*^,^*^7AV^* and pUC19-*srs2^K41A,^*^∆^*^SIM^*, respectively. pUC19-*srs2^L844A^* or pUC19-*srs2^K41A^*^,^*^L844A^* was constructed from pUC19-*SRS2* or pUC19-*srs2^K41A^* by site-directed mutagenesis to substitute leucine-844 with alanine. pUC19-*srs2*^∆^*^783-998^*, pUC19-*srs2*^∆^*^875-902^*, pUC19-*srs2^L844A^*^,∆^*^875-902^*, pUC19-*srs2^K41A^*^,∆^*^783-998^*, pUC19-*srs2^K41A^*^,∆^*^875-902^* or pUC19-*srs2^K41A^*^,^*^L844A^*^,∆^*^875-902^* were constructed from pUC19-*SRS2*, pUC19-*srs2^L844A^*, pUC19-*srs2^K41A^* or pUC19-*srs2^K41A^*^,^*^L844A^* by inverse PCR. Plasmid constructs for *srs2^K41A^*^,^*^3KR^* or *srs2^K41A^*^,^*^7AV^*^,^*^3KR^* were obtained by replacing the *Nde*I-*Bgl*II fragment of pUC19-*srs2^K41A^* or pUC19-*srs2^K41A^*^,^*^7AV^* with the *Nde*I-*Bgl*II fragments containing *3KR* mutations from pUC19-*srs2^3KR^*, resulting in pUC19-*srs2^K41A^*^,^*^3KR^* and pUC19-*srs2^K41A^*^,^*^7AV^*^,^*^3KR^*, respectively. For construction of a single copy plasmid bearing wild-type *SRS2* or its mutants, the *Bam*HI fragments containing the promoter and their coding regions from the pUC19-*SRS2* derivatives were cloned into pRS415. Expression constructs for wild-type Srs2 or its mutant proteins were constructed by PCR amplification of the protein coding region from the pUC-derived plasmid, and cloning of the amplified DNA fragment into galactose-inducible vector, p415GAL1. For their expression from the *AUR1* locus, the *Sph*I-*Kpn*I fragments of p415GAL1 derivatives bearing wild-type *SRS2* or its mutants were separately cloned into the integration vector, pAUR101 (Takara). The resulting plasmids were linearized at a unique *Stu*I site within the *AUR1* sequence of pAUR101 and introduced into the *AUR1* locus. The DNA sequences of the PCR-amplified fragments were confirmed by sequencing the appropriate regions.

**References**

1. Hishida T, Hirade Y, Haruta N, Kubota Y, Iwasaki H (2010) Srs2 plays a critical role in reversible G2 arrest upon chronic and low doses of UV irradiation via two distinct homologous recombination-dependent mechanisms in postreplication repair-deficient cells. Mol Cell Biol 30: 4840-4850.

2. Kolesar P, Sarangi P, Altmannova V, Zhao X, Krejci L (2012) Dual roles of the SUMO-interacting motif in the regulation of Srs2 sumoylation. Nucleic Acids Res 40: 7831-7843.

3. Pfander B, Moldovan GL, Sacher M, Hoege C, Jentsch S (2005) SUMO-modified PCNA recruits Srs2 to prevent recombination during S phase. Nature 436: 428-433.

4. Liberi G, Chiolo I, Pellicioli A, Lopes M, Plevani P, et al. (2000) Srs2 DNA helicase is involved in checkpoint response and its regulation requires a functional Mec1-dependent pathway and Cdk1 activity. Embo J 19: 5027-5038.

5. Chiolo I, Carotenuto W, Maffioletti G, Petrini JH, Foiani M, et al. (2005) Srs2 and Sgs1 DNA helicases associate with Mre11 in different subcomplexes following checkpoint activation and CDK1-mediated Srs2 phosphorylation. Mol Cell Biol 25: 5738-5751.
